# Steric control of redox events in organo-uranium chemistry: synthesis and characterisation of U(v) oxo and nitrido complexes†

**DOI:** 10.1039/c6sc00632a

**Published:** 2016-04-11

**Authors:** Nikolaos Tsoureas, Alexander F. R. Kilpatrick, Christopher J. Inman, F. Geoffrey N. Cloke

**Affiliations:** a School of Life Sciences, Division of Chemistry, University of Sussex Falmer Brighton BN1 9QJ UK f.g.cloke@sussex.ac.uk; b Chemistry Research Laboratory, Department of Chemistry, University of Oxford 12 Mansfield Road OX1 3TA Oxford UK

## Abstract

The synthesis and molecular structures of a U(v) neutral terminal oxo complex and a U(v) sodium uranium nitride contact ion pair are described. The synthesis of the former is achieved by the use of ^*t*^BuNCO as a mild oxygen transfer reagent, whilst that of the latter is *via* the reduction of NaN_3_. Both mono-uranium complexes are stabilised by the presence of bulky silyl substituents on the ligand framework that facilitate a 2e^−^ oxidation of a single U(iii) centre. In contrast, when steric hindrance around the metal centre is reduced by the use of less bulky silyl groups, the products are di-uranium, U(iv) bridging oxo and (anionic) nitride complexes, resulting from 1e^−^ oxidations of two U(iii) centres. SQUID magnetometry supports the formal oxidation states of the reported complexes. Electrochemical studies show that the U(v) terminal oxo complex can be reduced and the [U(iv)O]^−^ anion was accessed *via* reduction with K/Hg, and structurally characterised. Both the nitride complexes display complex electrochemical behaviour but each exhibits a quasi-reversible oxidation at *ca.* −1.6 V *vs.* Fc^+/0^.

## Abstract 2

Controlling the steric environment in U(η^8^-C_8_H_6_(1,4-SiR_3_)_2_)(η^5^-Cp*)] enables selective formation of either mononuclear U(v) or dinuclear U(iv) oxo and nitrido complexes.

## Graphical abstract

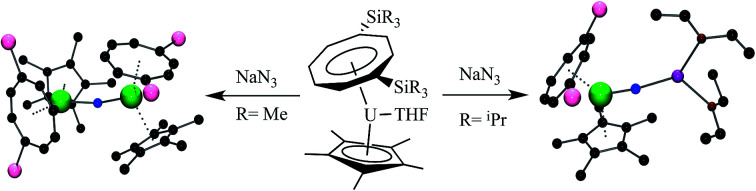

## Introduction

The study of well-defined molecular complexes of uranium is a thriving field of research,^1^ with significant current interest in the activation of small molecules and organic substrates by U(iii) compounds,^2^ the stabilisation of low oxidation states (*i.e.* U(ii)^3^) and also the study of higher oxidation state complexes featuring U⋯E (E = main group element) multiple bonds.^4^ Historically, complexes featuring U⋯O terminal bonds have been dominated by the ubiquitous uranyl moiety,^5^ partly due to its apparent chemical inertness (although recently disproved^6^) and its technological relevance to the nuclear cycle.^7^ In contrast, terminal mono-oxo complexes are much less common partly due to the increased nucleophilicity of the oxo ligand,^4*u*,8^ which leads to the formation of dimeric species,^8*a*,9^ and stabilisation of monomeric U=O complexes requires the use of bulky supporting ligands.^4*o*,10,11,12^ The relative rarity of uranium terminal oxo complexes is paralleled by the case of terminal nitride uranium complexes,^13,14^ and the majority of unsupported U⋯N bonds are stabilised in dimeric/polymeric structures.^15^ Indeed, until 2012 no stable, well-defined uranium terminal nitride complex was known,^13^ although UN triply bonded species had been spectroscopically identified in low temperature matrices,^16^ and *in situ* generation and involvement in C–H activation had been proposed and studied computationally.^17^

We have previously demonstrated the significance of the steric environment around the uranium centre in controlling the reductive coupling of CO_2_(ref. 18) and CO,^19^ promoted by U(iii) mixed sandwich complexes of the general type [U{η^8^-C_8_H_6_-(1,4-SiR_3_)_2_}(η^5^-Cp^R′′^)THF] (R = ^i^Pr (1), Me (2)). In particular, the reductive transformations (*i.e.* coupling, disproportionation, or reduction) of CO_2_ using the complexes [U{η^8^-C_8_H_6_-(1,4-SiMe_3_)_2_}(η^5^-Cp^Me_4_R′^)THF] (A) can be largely controlled by varying the size of R′ (R′ = Me, Et, ^i^Pr, ^*t*^Bu).^18*a*^ Unlike complexes of type A that exhibit a clear trend between the effect of steric environment and the outcome of the possible reductive transformations, when the analogous complexes in which the SiMe_3_ group had been replaced by the bulkier Si^i^Pr_3_ group were reacted with CO_2_, either intractable reaction mixtures were obtained or the reductive disproportionation of CO_2_ was promoted exclusively.^18*b*^ In order to better understand this observation, we envisaged that a study of the reactivity towards other heteroallenes (*e.g.* RN=C=O) as model substrates for CO_2_ might be informative.^20^

## Results and discussion

**Fig. 1:**
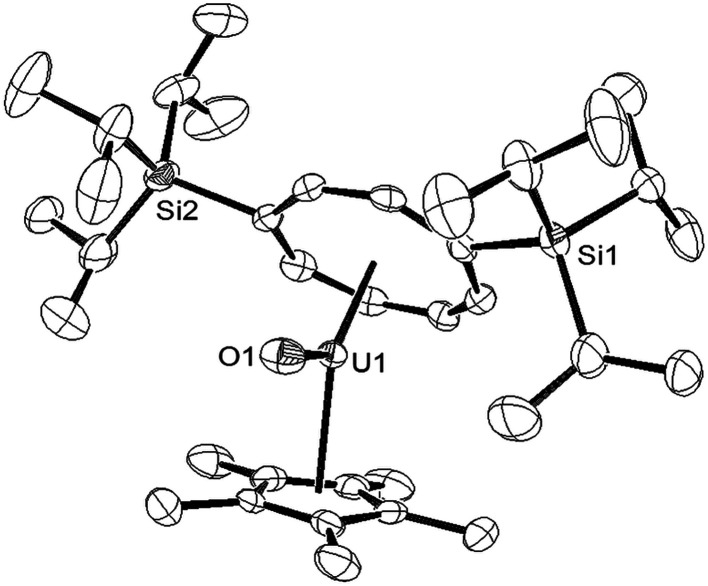
ORTEP-3 diagram of the molecular structure of (3) displaying 50% probability ellipsoids. H atoms have been omitted for clarity. Selected bond-lengths (Å) and angles (°): U1–O1 1.826(3), Ct(COT)–U 1.949(5), Ct(Cp*)–U1 2.492(1); Ct(COT)–U1–Ct(Cp*) 135.27(2), Ct(COT)–U1–O1 162.24(7), Ct(Cp*)–U1–O1 108.08(2).

Reaction of a brown-olive green C_6_D_6_ solution of [U{η^8^-C_8_H_6_-(1,4-Si^i^Pr_3_)_2_}(η^5^-Cp*)THF] (1) with a slight excess (1.05–1.1 eq.) of ^*t*^BuN=C=O under an Ar atmosphere resulted in an immediate colour change to brown red. ^1^H-NMR spectroscopy showed complete consumption of (1) and the formation of a new uranium species and free ^*t*^BuNC (further confirmed by GC-MS of the trapped volatiles of the reaction mixture). The ^29^Si{^1^H}-NMR spectrum of the product displayed a single resonance at −73 ppm, shifted downfield from −129 ppm in (1) suggesting that a change in the formal oxidation state of the uranium centre of (III) to (V) had taken place, in accordance with the general trend observed by Evans *et al.*^21^ The mass spectrum was consistent with the formation of the U(v) terminal oxo complex {U[η^8^-C_8_H_6_(1,4-Si^i^Pr_3_)_2_](η^5^-Cp*)O} (3), and was confirmed by X-ray crystallography (Fig. 1).

The U–O bond length in (3) (1.826(3) Å) is shorter than that found in the U(v) terminal oxo complex [Ph_3_PMe][U(O)(CH_2_SiMe_2_NR′)(NR′_2_)_2_] (1.847(2) Å),^22^ but similar within esd's to those in the U(v) complexes [U(O)(NR′_2_)_3_] (1.817(1) Å),^10^ [UTREN^TIPS^(O)] (1.856(6) Å, TREN^TIPS^ = [N(CH_2_CH_2_NSi^i^Pr_3_)_3_]^3^),^11*a*^ [((^R^ArO)tacn)U(O)] (1.848(8) Å; R = ^*t*^Bu, Ad; tacn = triazacyclononane),^4*o*^ Cp*_2_U(O)(ODipp) (1.859(6) Å, Dipp = 2,6-^i^Pr_2_-C_6_H_3_),^23^ [OU{OSi(O^*t*^Bu)_3_}_4_K] (1.825(2) Å)^12^ and [NEt_4_][*trans*-U(NR_2_′)_3_(O)CN].^24^ The Ct(COT)–U–Ct(Cp*) angle of 135.27(2)° is significantly more acute than those found in U(iv) (137–140°) and U(iii) (150–155°) mixed sandwich complexes supported by these ligands;^4*v*,18*a*,19*c*,25^ the reason for this is not clear but one possible explanation could be to minimise electrostatic repulsion between the anionic ligand and the polarised U–O bond. Compound (3) was further characterised by spectroscopic^26^ and analytical techniques (see ESI†), and Evans method (C_7_D_8_) gave an effective magnetic moment (*μ*_eff_) of 2.49 *μ*_B_, very close to the theoretical value of 2.54 *μ*_B_ for an f^1^ system (see below for further details and SQUID magnetometry).

**Fig. 2:**
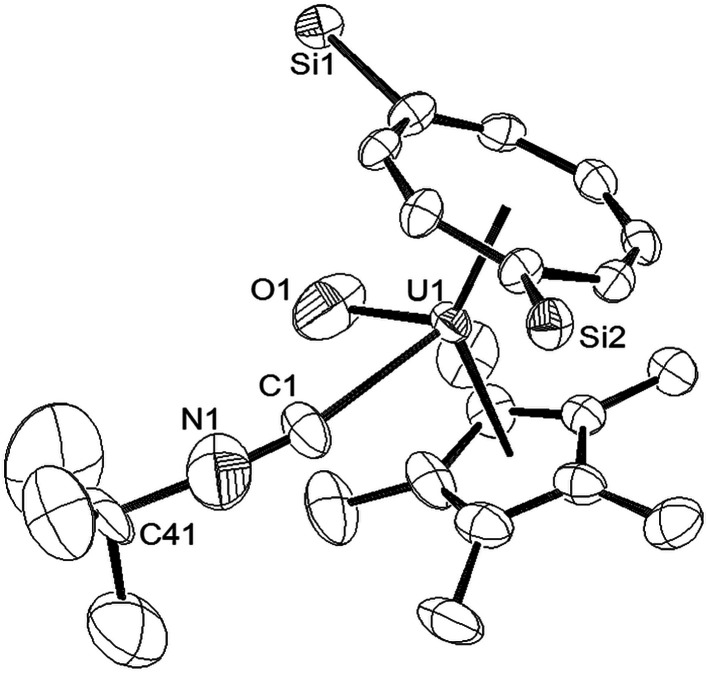
ORTEP-3 diagram of the molecular structure of (4) displaying 50% probability ellipsoids. H atoms and ^i^Pr groups have been omitted for clarity. Selected bond-lengths (Å) and angles (°): U1–C1 2.545(8), C1–N1 1.221(12), U1–O1 1.916(8) C41–N1 1.492(13) Ct(COT)–U1 2.005(2), Ct(Cp*)–U1 2.503(1); N1–C1–U1 162.8(9), C1–N1–C41 169.9(12), O1–U1–C1 71.0(4), Ct(COT)–U1–Ct(Cp*) 137.19(2), Ct(COT)–U1–O1 118.85(2), Ct(Cp*)–U1–O1 95.30(2), Ct(COT)–U1–C1 119.21(8), Ct(Cp*)–U1–C1 94.83(3).

When the synthesis of (3) was repeated on a larger scale, a second species co-crystallised with (3), and fractional crystallisation produced a small crop of crystals suitable for single crystal X-ray diffraction. The latter revealed the product to be the ^*t*^BuNC adduct of (3) [U(η^8^-C_8_H_6_{1,4-Si^i^Pr_3_}_2_)(η^5^-Cp*)O(η^1^-CN^*t*^Bu)] (4), and the molecular structure is shown in Fig. 2.

The most salient feature of (4) is the elongation of the U–O bond by almost 0.1 Å as compared with that in (3), and also with the U^(V)^=O bonds compared above, with the exception of that in [U(O)(NR′_2_)_3_] (R′ = SiMe_3_).^10^ The reason for this structural feature is unclear, but a possible explanation could be that the isocyanide ligand acts predominantly as a σ-donor with the extra electron density transferred to π symmetry orbitals of the uranium centre involved in antibonding contributions to the U–O bond. IR spectroscopy (*vide infra*) revealed *ν*_NC_ at 2179 cm^−1^ for the isocyanide ligand in (4), a value very close to those observed in [UCp*_2_(NMe_2_)(^*t*^BuNC)_2_]BPh_4_ (ref. 27) and the [UCp_3_(CNC_6_H_11_)(NCMe)]^+^ cation;^28^ the short (1.221(12) Å) CN bond in (4) is also comparable (within esd's) to those in the latter complexes, while the small deviation of the C–N–C(^*t*^Bu) from linearity presumably alleviates steric congestion around the metal centre. The Ct(COT)–U1–Ct(Cp*) angle is slightly more obtuse (*ca.* 2°) than that in (3), while the Ct(Cp*)–U1 and Ct(COT)–U1 distances are slightly elongated but within the range observed for previously reported complexes supported by these ligands.

Attempts to isolate (4) in better yields from the reaction of (1) with ^*t*^BuNCO were unsuccessful, leading to mixtures of (3) and (4), and indeed the ^*t*^BuNC ligand in (4) is very labile and any attempted isolation or manipulation of (4) *via* operations *in vacuo* invariably again led to mixtures of (3) and (4). In order to isolate (3) free from (4), the best route involved the reaction of (1) with ^*t*^BuNCO followed by repeated dissolution in pentane and subsequent evaporation, a method used by Andersen and Evans *et al.* to obtain base-free Cp* lanthanide complexes,^29^ which afforded (3) in 55% yield. Reaction of a C_6_D_6_ solution of the resultant microanalytically pure (3) with one equivalent of ^*t*^BuNC resulted in small but discernible shifts of the resonances due to (3) in the ^1^H-NMR spectrum and which we ascribe to the formation of (4). Similarly, *in situ* IR spectroscopy showed that, upon reaction of (3) with 1 equivalent of ^*t*^BuNC in methyl–cyclohexane, two new peaks appeared, one at 2134 cm^−1^ (*ν*_NC_ in free ^*t*^BuNC) and one at 2179 cm^−1^ assigned to *ν*_NC_ in (4).

**Scheme 1:**
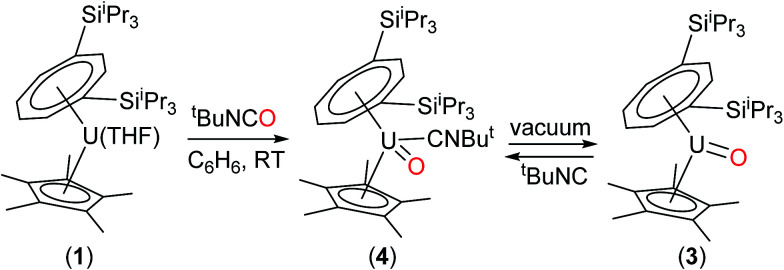
Synthesis of terminal oxo U(v) complexes (3) and (4).

The above data suggest that the synthesis of the novel U(v) terminal oxo complex (3) proceeds *via* the isocyanide adduct (4): the use of ^*t*^BuNCO as an efficient oxygen transfer reagent^30^ results in the two electron oxidation of (1) and the formation of ^*t*^BuNC and hence (4) (probably *via* a concerted reaction), and ultimately (3) after work-up (Scheme 1).

**Scheme 2:**
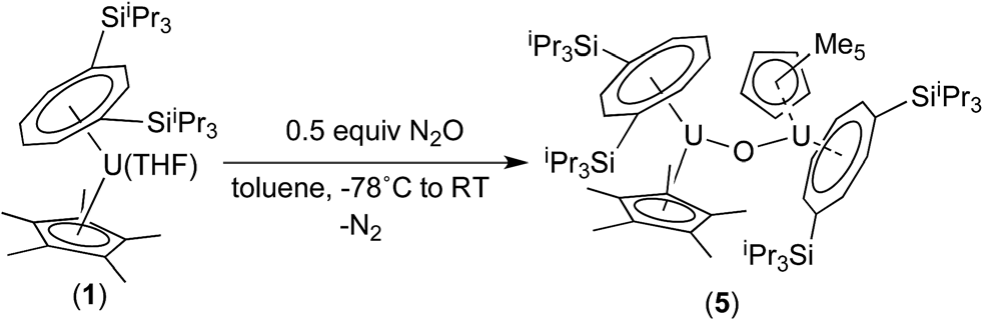
Synthesis of (5).

We have previously reported the synthesis of the dimeric, μ-oxo U(iv) complex {U[η^8^-C_8_H_6_(1,4-Si^i^Pr_3_)_2_](η^5^-Cp*)}_2_(μ-O) (5) from the reaction of (1) with a mixture of NO/CO.^31^ Given the existence of (5), the isolation of the mononuclear terminal oxo U(v) complex (3) would appear surprising. We therefore decided to investigate whether (3) could be prepared using alternative oxygen transfer reagents. Reaction of (1) with exactly 0.5 equivalents of N_2_O (administered accurately *via* a Töepler line) in C_7_D_8_ at −78 °C resulted in an immediate colour change to bright red, leading to the clean formation of (5) as evidenced by ^1^H and ^29^Si{^1^H}-NMR spectroscopy, and the μ-oxo complex was isolated as the sole product in very good yields (see Scheme 2 and ESI†).

**Scheme 3:**
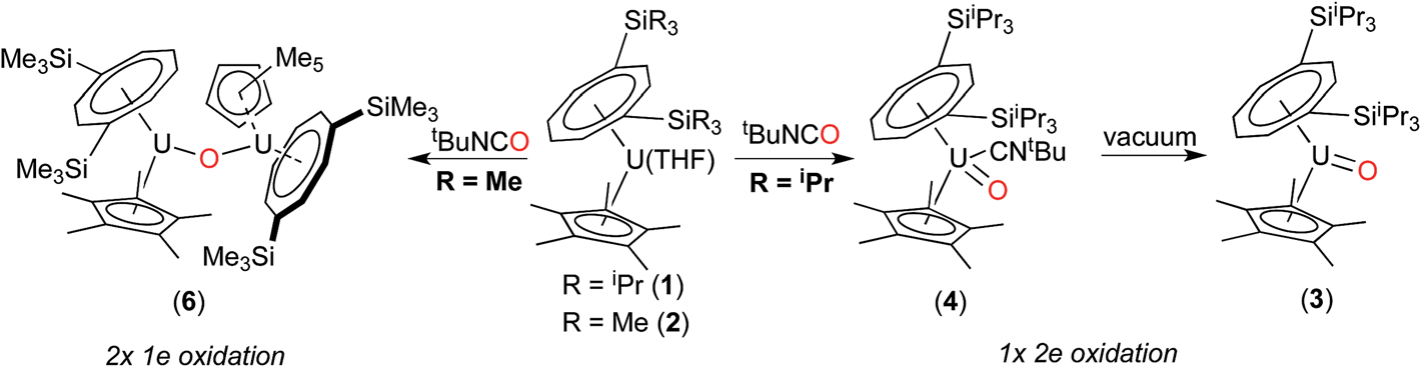
Steric control of the oxidation state of the U centre.

On the other hand, when equimolar amounts of U(v) terminal oxo complex (3) and the U(iii) precursor (1) were mixed in C_7_D_8_, no reaction was observed at RT and conversion to the μ-oxo complex (5) (*ca.* 25% spectroscopic yield relative to (1)) was observed only after heating at 45 °C over three days.^32^ These experiments in conjunction with the isolation of (4) indicate that these two reactions most likely proceed *via* different mechanisms. The case of N_2_O would be consistent with a concerted mechanism involving a dinuclear intermediate in which N_2_O bridges, and then eliminates N_2_ leading to a dinuclear μ-oxo product. However for ^*t*^BuNCO, the formation of mononuclear (4) after the oxo transfer step, stops any further reaction with (1) that could lead to (5), due to the steric congestion imposed by both the TIPS groups and the ^*t*^BuNC ligand. To further test this hypothesis, the less sterically hindered homologue of (1), [U{η^8^-C_8_H_6_-(1,4-SiMe_3_)_2_}(η^5^-Cp*)THF] (2) was reacted with ^*t*^BuNCO. In this case the reaction furnished cleanly the dinuclear μ-oxo U(iv) complex {U[η^8^-C_8_H_6_(1,4-SiMe_3_)_2_](η^5^-Cp*)}_2_(μ-O) (6) as evidenced by its NMR spectroscopic data that were in excellent agreement with those previously reported.^18*a*^ Compounds (1) and (2) have very similar [U^(III)^] ↔ [U^(IV)^] redox potentials (−2.13 V and −2.10 V *vs.* Fc^+/0^ respectively, see ESI†), so the clean formation of (6) highlights the importance of the steric hindrance imposed by the silyl substituents on the 8-membered ring in dictating the outcome of the reactions of (1) and (2) with ^*t*^BuNCO. In the case of (1), reaction with ^*t*^BuNCO results in a single 2e^−^ oxidation of the metal centre leading to the U(v) complex (4), and hence (3), whereas in the case of (2) this reaction results in two 1e^−^ oxidations leading to the dinuclear U(iv)–U(iv) complex (6) (Scheme 3).

Attempts to generate (3) using other isocyanates (PhNCO, ^i^PrNCO) or oxo transfer reagents (Me_3_NO, pyridine *N*-oxide) were uniformly unsuccessful leading to intractable reaction mixtures. Interestingly when Me_3_SiNCO was reacted with (1), the U(iv) complex {U[η^8^-C_8_H_6_(1,4-Si(^i^Pr)_3_)_2_](η^5^-Cp*)(OSiMe_3_)} (7) was isolated as the sole product of the reaction.^33^ The isolation of (7) can reasonably be explained by the formation of a short-lived [U^(V)^=O] complex which, due to the oxophilicity of the SiMe_3_ group, undergoes a formal reduction to produce the observed U(iv) complex (7) and presumably cyanogen (CN)_2_ (although formation of the latter was not confirmed). Similar reactivity of U=O bonds towards silicon electrophiles has been observed by Andersen *et al.*^8*a*^

Given the similarities between nitride and oxo ligands,^14^ the successful isolation of the terminal oxo complex (3) suggested that the steric protection afforded by the U[η^8^-C_8_H_6_(1,4-Si^i^Pr_3_)_2_](η^5^-Cp*) mixed sandwich framework might be exploited to access the analogous uranium nitride. The highly reducing nature of (1) (U^III^/U^IV^ −2.13 V *vs.* Fc^+/0^), suggested reduction of N_3_^−^ as a possible method for installing the nitride ligand.^13*a*^

**Scheme 4:**
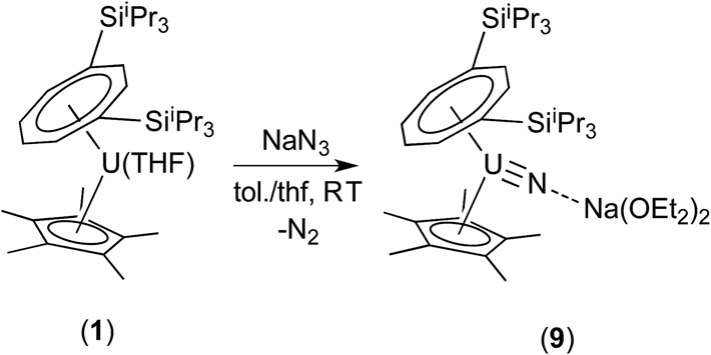
Synthesis of (9).

**Fig. 3:**
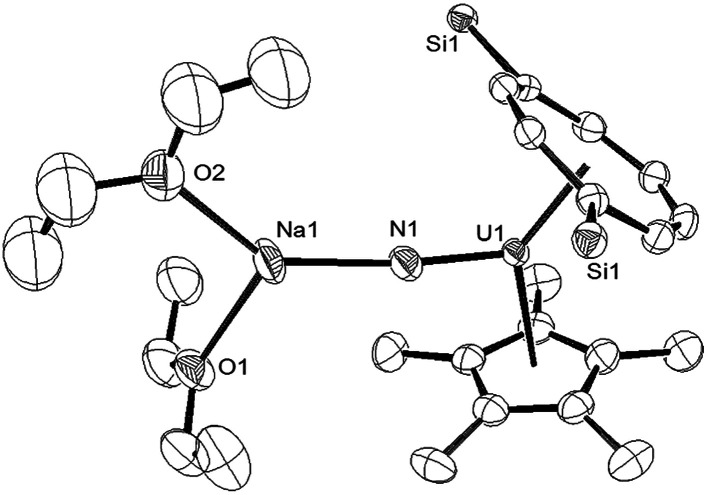
Ortep-3 diagram of the molecular structure of (9) displaying 50% probability ellipsoids. H atoms and ^i^Pr groups have been removed for clarity. Selected bond-lengths (Å) and angles (°): U1–N1 1.835(5), N1–Na1 2.244(6), Ct(COT)–U1 2.026(1), Ct(Cp*)–U1 2.548(8); U1–N1–Na1 172.4(3), Ct(COT)–U1-Ct(Cp*) 137.25(7), Ct(COT)–U1–N1 124.73(5), Ct(Cp*)–U1–N1 101.99(1).

Reaction of (1) with NaN_3_ (Scheme 4) in a mixture of C_7_H_8_/C_4_H_8_O resulted in a slow colour change to brown-red and after work-up and re-crystallisation from Et_2_O, brown crystals of (9) were isolated in moderate yield (*ca.* 30%), together with other product(s) which could not be unambiguously characterised despite repeated attempts. X-ray diffraction studies showed (9) to be the nitride complex [U{η^8^-C_8_H_6_-(1,4-Si^i^Pr_3_)_2_}(η^5^-Cp*)(μ-N)(μ-Na{OEt_2_}_2_)], best described as a sodium uranium nitride contact ion pair (Fig. 3).

Liddle *et al.* recently described a U(v) terminal nitride anion supported by the TREN^TIPS^ ligand, as well as its U(vi) neutral analogue.^13*a*,*b*^ The U–N bond length of 1.835(5) Å in (9) is comparable to that in the U(v) nitride complex [U(TREN^TIPS^)N]^−^[Na(12-c-4)_2_]^+^ (1.825(15) Å) where the two ions are separated, but is shorter than the one found in [U(TREN^TIPS^)(μ-N)(μ-Na)]_2_ (1.883(4) Å) where a N–Na interaction is also present.^13*a*^ It is also shorter than those in the borane capped nitrido complexes [(C_6_F_5_)_3_BNU^(V)^(NMes^*t*^Bu)_3_][N^*n*^Bu] (1.916(4) Å) and [(C_6_F_5_)_3_BNU^(VI)^(NMes^*t*^Bu)_3_] (1.880(4) Å)^34^ (although the latter two can viewed as borane–imido complexes and the bond distances are more typical of U imido complexes). Compared to the neutral U(vi) complex [U(TREN^TIPS^)N] the U–N bond in (9) is similar within esd's.^13*b*^ The Na–N bond length of 2.244(6) Å in (9) is shorter than the ones found in [U(TREN^TIPS^)(μ-N)(μ-Na)]_2_ (2.308(5) Å)^13*a*^ and [U(TREN^TIPS^)(μ-N)(μ-Na{15-c-5})] (2.291(5) Å),^13*b*^ and the U–N–Na linkage is close to linear as in the latter. The Ct(Cp*)–U distance in (9) is elongated compared to (3) and (4) while the Ct(COT)–U1–N1 and Ct(Cp*)–U1–N1 angles are significantly more acute than the ones found for the corresponding angles Ct–U1–O1 angles in (3). The reason for these differences is unclear.

Complex (9) was further characterised by spectroscopic^35^ and analytical techniques (see ESI†), and the *μ*_eff_ (Evans method) was determined to be 2.21 *μ*_B_ (further details below, including SQUID magnetometry), which is in reasonable agreement with the value of 1.99 *μ*_B_ for [U(TREN^TIPS^)N]^−^,^13*a*^ and is within the range of values reported for other U(v) complexes.^36^

The ^23^Na NMR spectrum of (9) in THF revealed a single, very broad (Δ*ν*_1/2_ = 8300 Hz) resonance centred at *ca. δ* 200 ppm suggesting that the interaction of the sodium cation with the paramagnetic uranium centre is maintained in solution (*cf.* (10), *vide infra*).

**Scheme 5:**
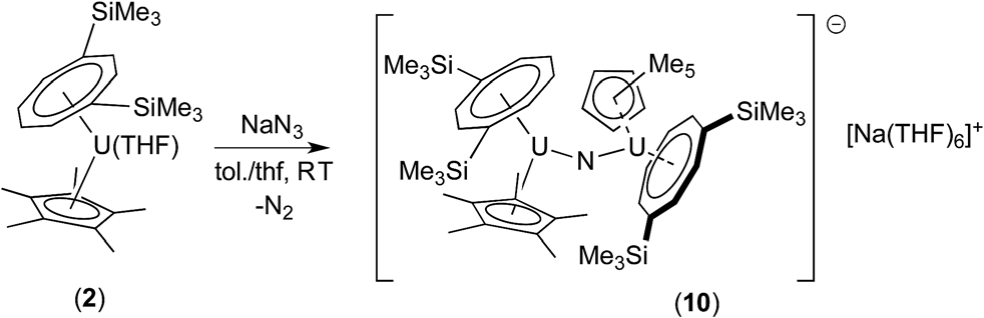
Synthesis of (10).

**Fig. 4:**
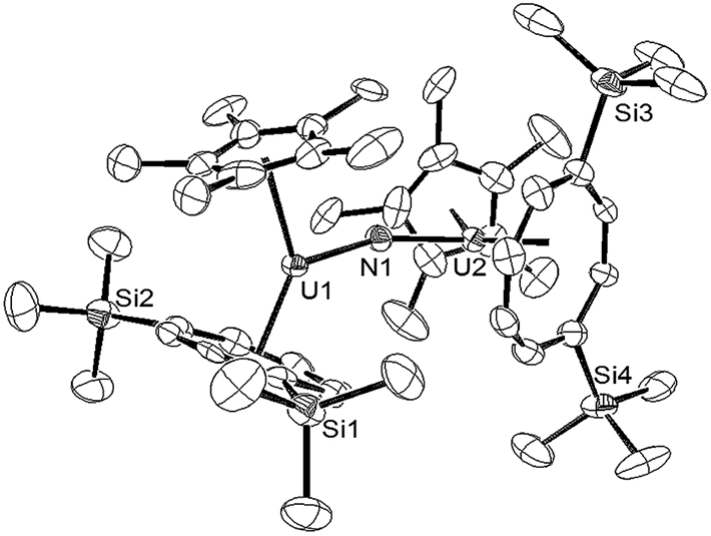
ORTEP-3 diagram of the molecular structure of the anion in (10) displaying 50% probability ellipsoids. H atoms have been removed for clarity. Selected bond-lengths (Å) and angles (°): N1–U1 2.063(5), U2–N1 2.066(5), Ct(COT)–U1 2.033(4), Ct(Cp*)–U1 2.516(2), Ct(COT)–U2 2.038(4), Ct(Cp*)–U2 2.536(2); U1–N1–U2 159.4(3), Ct(COT)–U1–Ct(Cp*) 137.03(2), Ct(COT)–U2–Ct(Cp*) 136.70(2), Ct(COT)–U1–N1 122.83(8), Ct(Cp*)–U1–N1 100.09(1), Ct(COT)–U2–N1 122.75(3), Ct(Cp*)–U2–N1 100.54(2).

Since the less sterically hindered U(iii) complex [U{η^8^-C_8_H_6_-(1,4-SiMe_3_)_2_}(η^5^-Cp*)THF] (2) affords the bridging μ-oxo complex (6), the reaction of (2) with a slight excess of NaN_3_ (1.5 mol eq.) in a C_7_H_8_/THF solvent mixture (*ca.* 2 : 1) was explored. Indeed, after work-up and re-crystallisation from THF/Et_2_O, brown-red crystals of the bridging nitride complex [{U[η^8^-C_8_H_6_(1,4-SiMe_3_)_2_](η^5^-Cp*)}_2_(μ-N)]^−^[Na(THF)_6_]^+^ (10) suitable for X-ray diffraction studies were isolated in 81% yield (Scheme 5 and Fig. 4).

The two U–N bond lengths (N1–U1 2.063(5) Å, U2–N1 2.066(5) Å) in the anionic dimer are essentially the same, suggesting a delocalised [U ≃ N ≃ U] bonding interaction as in [Cp*_2_U(μ-N)(μ-N_3_)UCp*_2_]_4_,^15*e*^ and also the same within esd's to the ones previously reported for other bridging nitride complexes with the exception of the triply bridging nitride in [{UCp*_2_(μ-I)_2_}_3_(μ_3_-N)] (2.152(3)–2.138(3) Å).^15*d*^ Furthermore the length of the bond is in the middle of the range found for complexes with localised U–N=U bonding interactions (1.95–2.12 Å).^15*b*,*c*^ Compared to the U–N bond length in (9), that in (10) is significantly elongated as expected. The U–N–U bond in (10) has significantly deviated from linearity, which is a common structural motif for many bridging U nitride complexes^15*f*,37^ but is less obtuse than those in the [U^(IV)^–N–U^(IV)^]^−^, [U^(IV)^–N–U^(V)^] and [U^(IV)^–N–U^(VI)^(O)]^−^ complexes supported by bulky silyl amide ligands,^15*c*^ that in [KU(μ-N)(OSi(O^*t*^Bu)_3_)]_2_ (106.1(2)°),^15*b*^ as well as in complexes where the nitride ligand bridges more than two U centre.^15*a*,*d*,*f*^ Unsurprisingly, the U–N–U bond angle in (10) is identical to the U–O–U bond angle found in the μ-oxo complex {U[η^8^-C_8_H_6_(1,4-SiMe_3_)_2_](η^5^-Cp*)}_2_(μ-O) (6)^18*a*^ – a fact that reflects the effect of the sterically imposed geometry of the complex. As expected the U–N bonds are shorter than the corresponding U–O ones in (6) and that shortening might account for the slightly more acute Ct(COT)–U–Ct(Cp*) angles in (10) compared to the ones found in (6) (139.7(16)° and 140.0(16)°).^18*a*^

Complex (10) readily loses its crystallinity due to loss of coordinated THF to yield [{U[η^8^-C_8_H_6_(1,4-SiMe_3_)_2_](η^5^-Cp*)}_2_(μ-N)]^−^[Na(THF)_2_]^+^ (10′) as a well-defined product, as evidenced by microanalysis. As in the case of its μ-oxo analogue [{U[η^8^-C_8_H_6_(1,4-SiMe_3_)_2_](η^5^-Cp*)}_2_(μ-O)] (6), the ^1^H-NMR (C_4_D_8_O_2_) spectrum of (10′) is consistent with a *C*_2_-symmetric structure that is retained in solution. In marked contrast to (9), the ^23^Na NMR spectrum of (10) in THF exhibited a sharp resonance (Δ*ν*_1/2_ = 78 Hz) at *δ* −7.94 ppm, parameters suggesting no interaction of the [Na(THF)_6_]^+^ counterion with the paramagnetic uranium anion.^38^

Similarly to the reaction of (2) with ^*t*^BuNCO that yields the μ-oxo complex (6), the bridging nitride complex (10) can be seen as the product of two 1e oxidations of the U(iii) precursor (*vs.* the one 2e oxidation that produces (9) in the case of the bulkier COT substituents), since the formal oxidation state of the uranium centres in (10) is +4. The *μ*_eff_ for 10′ (C_4_D_8_O_2_, Evans method) was determined to be 3.64 *μ*_B_ for the dimer or 2.57 *μ*_B_ per uranium centre, a value consistent with a U(iv) ion (further details including SQUID magnetometry below).

### Magnetic studies on (3), (9) and (10′)

Table 1 compares the *μ*_eff_ for complexes (3), (9) and (10′) at 300 K as determined in solution (Evans method), and in the solid state (SQUID under an applied field of 0.1 tesla); the values determined by these two methods are in fair agreement.

**Table 1:** *μ*_eff_ of (3), (9) and (10′) at 300 K in solution and the solid state

Complex	*μ*_eff_ Evans (*μ*_B_)	*μ*_eff_ SQUID (*μ*_B_)
(3)	2.49	2.16
(9)	2.2	2.00
(10′)	3.64 (2.57 per U)	3.58 (2.53 per U)

**Fig. 5:**
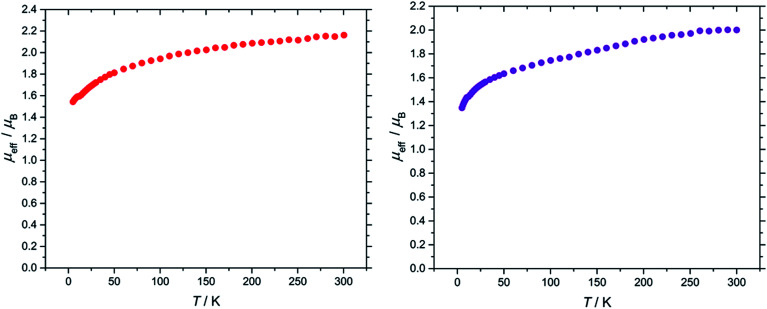
Temperature dependence of the solid state *μ*_eff_ of (3) (left) and (9) (right) at 0.1 tesla.

The effective magnetic moment of (3) exhibits a steady decline from the value of 2.16 *μ*_B_ at 300 K to 1.54 *μ*_B_ at 5 K (Fig. 5). This behaviour is typical for a ^2^F_5/2_ ion, and is comparable to values reported for molecular U(v) terminal oxo complexes (see ESI† for plots of *χ*_m_/*T*, *χ*_m_*T*/*T* and *χ*_m_^−1^/*T*).^4*o*,22,23^

In the case of the nitride complex (9), its effective magnetic moment was found to be 2.00 *μ*_B_ at 300 K and 1.35 *μ*_B_ at 5 K (Fig. 5). These values are comparable to the effective magnetic moment found for nitride complex [UN(TREN^TIPS^)][Na(12-crown-4)_2_] (1.99 *μ*_B_ at 298 K, 1.31 *μ*_B_ at 1.8 K),^13*a*^ and are in agreement with literature values for molecular U(v) complexes more generally^39^ (see ESI† for plots of *χ*_m_/*T*, *χ*_m_*T*/*T* and *χ*_m_^−1^/*T*).

**Fig. 6:**
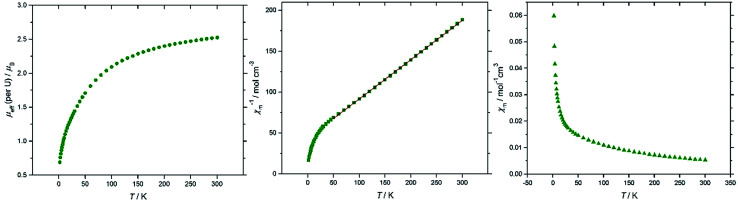
Magnetic data for (10′). From left to right: *μ*_eff_ (per U)/*T*; *χ*_m_^−1^/*T* (red line is a linear fit to the data in the range 50–300 K); *χ*_m_/*T* (see ESI† for the plot of *χ*_m_*T*/*T*).

Magnetic susceptibility data sets for (10′) measured for zero-field cooled and field cooled samples coincided exactly, indicating the absence of long–range interactions between spins on the two U(iv) centres. At 300 K the effective magnetic moment per U is 2.53 *μ*_B_, and decreases to 0.69 *μ*_B_ at 2 K (Fig. 6a), consistent with two U^IV^ f^2^ ions. For comparison, the solid state magnetic studies on the di-uranium(iv) dianion [{((^nP,Me^ArO)_3_tacn)U}_2_(μ-O)_2_]^2–^ by Meyer *et al.* showed a *μ*_eff_ per U of 2.73 *μ*_B_ at 300 K.^40^ The majority of paramagnetic substances have a molar susceptibility (*χ*_m_) that obeys the Curie–Weiss law, *χ*_m_ = *C*/(*T* − *Θ*), where *C* is the Curie constant and *Θ* is the Weiss constant. The plot of *χ*_m_^−1^*vs. T* (Fig. 6b) follows Curie–Weiss behavior in the range 50–300 K, with *C* = 0.0289 K^−1^ mol^−1^ and *Θ* = −0.015 K, suggesting that at these temperatures the [{U[η^8^-C_8_H_6_(1,4-SiMe_3_)_2_](η^5^-Cp*)}_2_(μ-N)]^−^ anion behaves as two non-interacting U^(IV)^ centres. Furthermore, there is no maximum observed in the *χ*_m_*vs. T* plot (Fig. 6c), often cited as a definitive indication of antiferromagnetic coupling. The U^(IV)^ ion (^3^H_4_ ground term) typically has minimal covalency, hence the two metal centres in 10′ do not participate in exchange coupling.

**Fig. 7:**
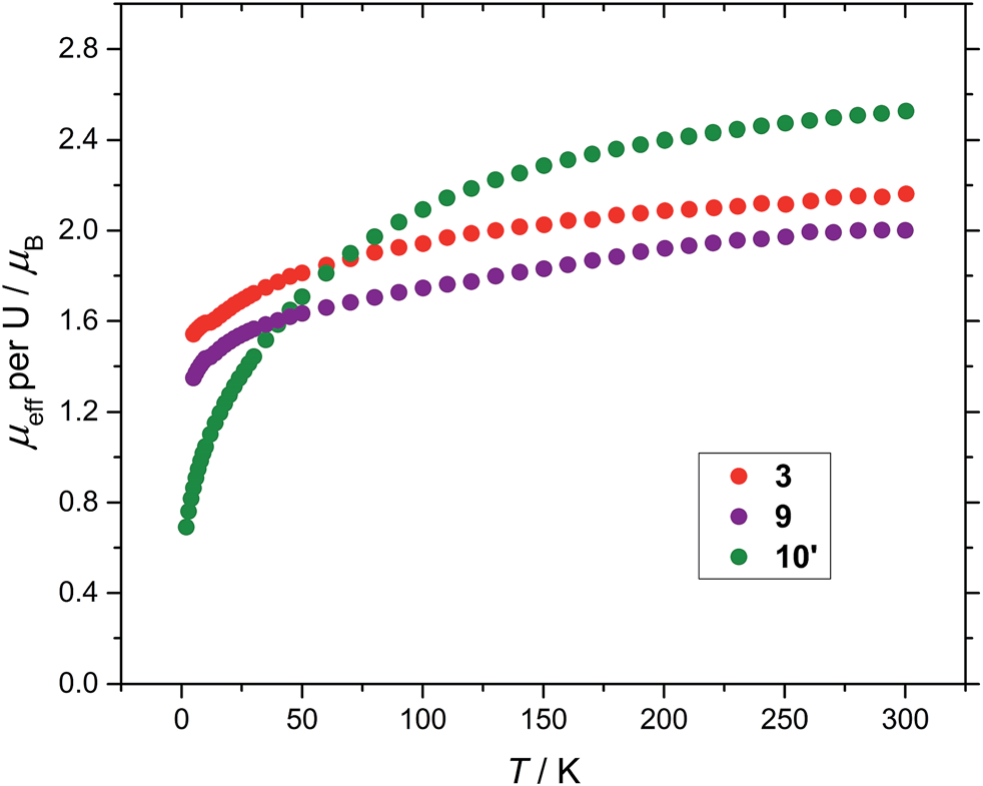
Temperature dependence of the solid state *μ*_eff_ of (3), (9) and (10′) (per U) at 0.1 tesla.

Finally, magnetic data for all three compounds (3), (9) and (10′) are presented in Fig. 7 for comparison.

### Redox behaviour of (3), (9) and (10)

In order to gauge the potential for accessing terminal oxo and nitrido uranium(vi) complexes, the redox properties of (3), (9) and (10) were studied by cyclic voltammetry (C.V.).

**Fig. 8:**
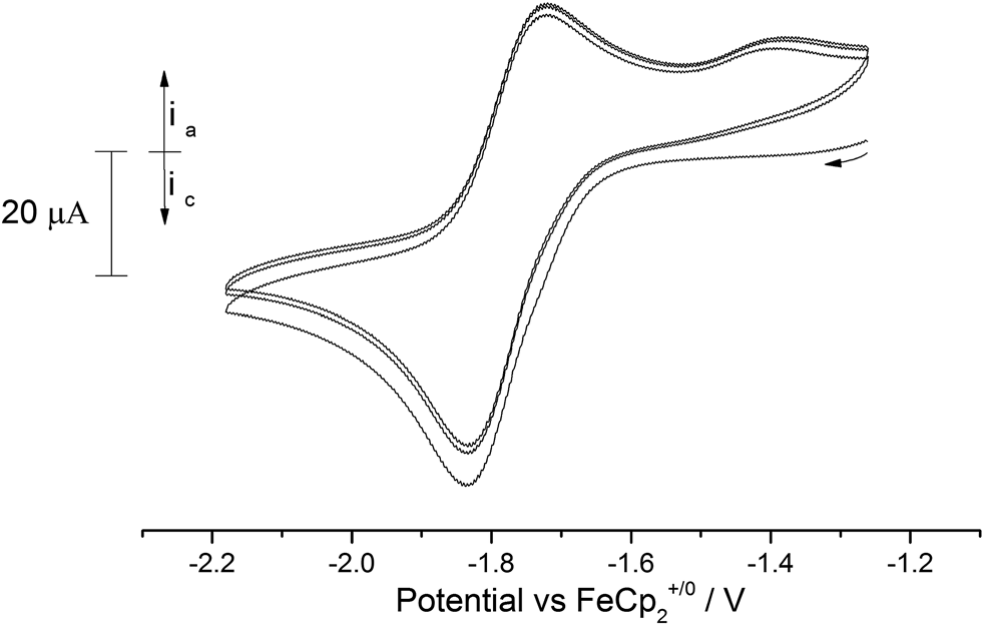
Overlaid CV scans (3 cycles) of (3) in 0.05 M [N(^*n*^Bu)_4_][B(C_6_F_5_)_4_]/THF. Scan rate 250 mV s^−1^.

In contrast to the terminal oxo [((^*t*Bu^ArO)tacn)U(O)] complex reported by Meyer *et al.* that features a reversible oxidation,^4*o*^ the C.V. of the terminal oxo complex (3) revealed only a quasi-reversible reduction process at −1.77 V *vs.* Fc^+/0^ (Fig. 8).

**Fig. 9:**
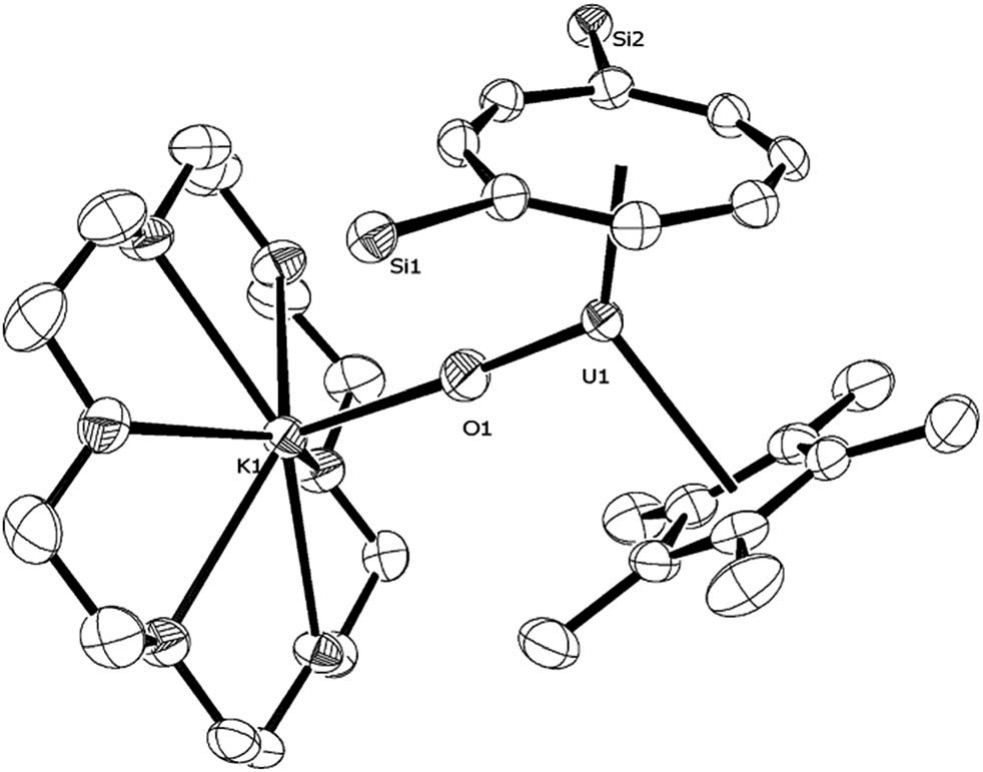
ORTEP-3 diagram of the molecular structure of (11) displaying 50% probability ellipsoids. H atoms, ^i^Pr groups and a molecule of toluene have been removed for clarity. Selected bond-lengths (Å) and angles (°): U1–O1 1.891(4), Ct(COT)–U 2.041(1), Ct(Cp*)–U1 2.583(4), O1–K1 2.582(4); U1–O1–K1 177.3(2), Ct(COT)–U1–Ct(Cp*) 133.81(2), Ct(COT)–U1–O1 125.73(6), Ct(Cp*)–U1–O1 100.45(2).

Changing the scan rate (50–300 mV s^−1^) did not alter the shape of the observed wave and no other processes were found to occur over the solvent window. This process is assigned to the [U^(V)^] ↔ [U^(IV)^] couple, and based on this voltammogram, the reduction of (3) should be a chemically accessible process. Indeed, (3) can be chemically reduced with a slight excess of K/Hg (0.5% w/w) in the presence of 18-crown-6 in *n*-pentane/Et_2_O. The almost instantaneous reaction produced a red-pink solid that, after work-up and re-crystallisation from toluene, gave dark red rods suitable for X-ray diffraction studies which showed the product to be the U(iv) complex [U{η^8^-C_8_H_6_-(1,4-Si^i^Pr_3_)_2_}(η^5^-Cp*)(μ-O)K(18-c-6)] (11) (Fig. 9).

The U–O bond length (1.891(4) Å) in (11) is longer than that in the U(v) complexes (3) (1.826(3) Å) and [U(NR_2_′)_3_O]^10^ (1.826(3) Å), but similar within esd's to the one found in the U(iv) complex (4) (1.916(8) Å). The K1–O1 bond length is as expected shorter than the O=U=O⋯K bonds (2.60–2.9 Å)^41^ and is typical of an ionic K–O bond;^12^ the U–O–K bond is very close to linear.

Complex (11) was fully characterised by spectroscopic and analytical methods (see ESI†); the ^29^Si{^1^H}-NMR was of particular diagnostic value as it was shifted upfield to −172.22 ppm (−72.7 ppm for parent (3)), a value that is even more upfield than that for the U(iii) complex (1) (−129 ppm), probably due to the anionic nature of (11).

**Fig. 10:**
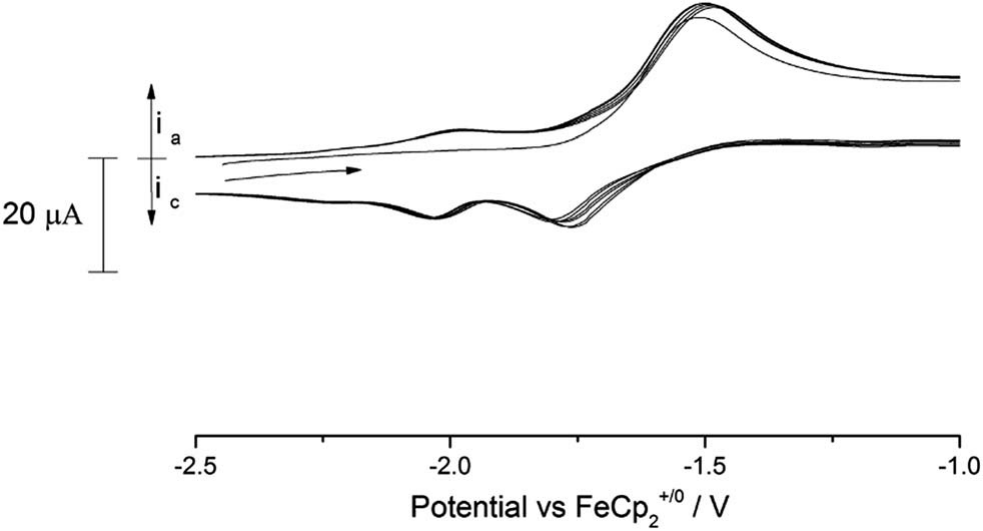
Overlaid scan (4 cycles) of (9) in 0.05 M [N(^*n*^Bu)_4_][B(C_6_F_5_)_4_]/THF. Scan rate 100 mV s^−1^.

C.V. scans of the nitride complex (9) in the anodic direction over several cycles revealed the existence of several processes in the accessible solvent window (see ESI Fig. SI8† for a full voltammogram). Of these processes, there is a noteworthy quasi-reversible oxidation at −1.63 V *vs.* Fc^+/0^ (Fig. 10) which we tentatively assign to the [U^(VI)^] ↔ [U^(V)^] couple. As can be seen from Fig. 10, a second process at slightly more cathodic potential (*ca.* −1.8 V *vs.* Fc^+/0^) is also present, which features an asymmetric current response that leads us to conclude that this is probably related to a short lived electrochemically generated species. The shape of the wave at −1.63 V did not change by variation of the scan rate (50–350 mV s^−1^).

In addition to this process, the (full) voltammogram of (9) exhibits also another two irreversible processes: one anodic at 0.7 V and a cathodic one at −2.8 V (both *vs.* Fc^+/0^). The nature of these two irreversible processes cannot be unambiguously assigned, but they could be due to ligand activation involving the nitride moiety. Attempts to chemically oxidise (9) by reaction with mild oxidants such as I_2_ and AgBPh_4_ have thus far resulted in intractable mixtures from which only ligand decomposition could be observed spectroscopically (^1^H-NMR).

**Fig. 11:**
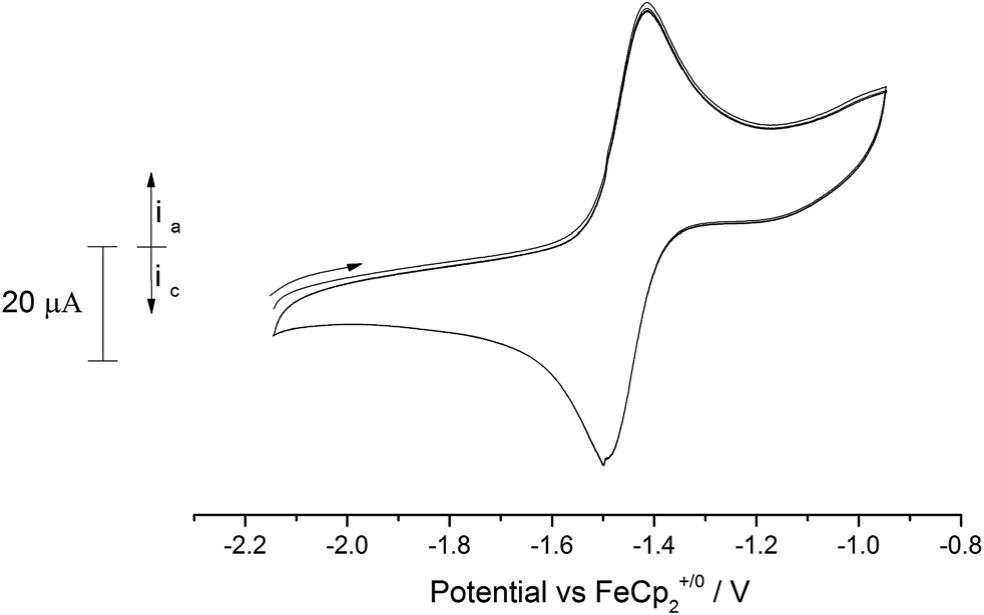
Overlaid CV scans (2 cycles) of (10′) in 0.1 M [N(^*n*^Bu)_4_][PF_6_]/acetonitrile. Scan rate 150 mV s^−1^.

Similarly, anodic scans of the bridging nitride (10) revealed a quasi-reversible process (peak separation 87 mV) centred at −1.46 V *vs.* Fc^+/0^ (Fig. 11). This value is very close to the one observed for complex (9) as well as for the {[U^(IV)^]=N=[U^(IV)^]}^−^ ↔ {[U^(V)^]=N=[U^(IV)^]} couple ([U] = U(NMes^*t*^Bu)_3_) reported by Cummins *et al.*^15*f*^ Based on this, we tentatively assign this process to the {[U^(IV)^]–N–[U^(V)^]} ↔ {[U^(IV)^]–N–[U^(IV)^]} redox pair.

Apart from this process, the voltammogram also displayed additional irreversible processes centred at anodic voltages (−0.5 V, −0.25 V, 0.35 V; see ESI Fig. SI10†) that are probably due to the formation of higher oxidation state (mixed valence) species (*i.e.* {[U^(V)^]–N–[U^(V)^]}, {[U^(VI)^]–N–[U^(V)^]} *etc.*), although other reasons (*e.g.* ligand activation) cannot be excluded. As in the case of (9) an irreversible reduction is also observed at *ca.* −2.5 V *vs.* Fc^+/0^ that as above could correspond to a mixed valence species (*i.e.* {[U^(III)^]–N–[U^(V)^]}) or arise from a ligand activation process. Given that similar processes appear in the case of (9), we envisage that they are more likely due to the latter rather than the former.

## Conclusion

In summary we have described how the steric environment around the metal centre can manipulate redox events at a uranium centre. This has been demonstrated by the isolation of either mononuclear U(v) or dinuclear U(iv) nitrido/oxo complexes depending on the size of the silyl substituents on the supporting ligands. This has led to the preparation of an anionic uranium(v) nitride complex (9) featuring a U–N triple bond, as well as a neutral U(v) terminal oxo complex (3). Magnetic studies corroborate the formal oxidation states of these complexes further confirming that the 2e^−^ oxidation leads to products featuring either one U(v) or two U(iv) metal centres depending on steric hindrance at the uranium centre. Cyclic voltammetry studies of complex (3) show that it can be readily reduced to the [U^(IV)^=O]^−^ anion (11), which has also been achieved chemically. Unlike (3), cyclic voltammetry studies have shown that the nitride complex (9) might be amenable to oxidation to the U(vi) species although initial attempts to do so have been unsuccessful thus far.

## Supplementary Material

SC-007-C6SC00632A-s001

SC-007-C6SC00632A-s002
