# Supplementary material for: Steric control of redox events in organo-uranium chemistry: synthesis and characterisation of U(v) oxo and nitrido complexes
Source: Chem Sci. 2016 Apr 11;7(7):4624–32. doi: 10.1039/c6sc00632a (PMC6013772; doi:10.1039/c6sc00632a)
Supplement: SC-007-C6SC00632A-s001 [file SC-007-C6SC00632A-s001.pdf]

Electronic Supplementary Material (ESI) for Chemical Science.
This journal is © The Royal Society of Chemistry 2016

Steric Control of Redox Events in Organo-uranium chemistry: Synthesis and Characterisation of U(V) Oxo and Nitrido complexes.

Nikolaos Tsoureas,[a] Alexander F.R. Kilpatrick,[b] Christopher J. Inman[a] and F. Geoffrey N. Cloke. [a*]

[a] *School of Life Sciences, Division of Chemistry, University of Sussex, Falmer, Brighton, BN1 9QJ, U.K.*

[b]*Chemistry Research Laboratory, Department of Chemistry, University of Oxford, 12 Mansfield Road, OX1 3TA, Oxford, U.K.*

Electronic Supplementary Information

Experimental:

General considerations: All manipulations were carried out in a MBraun glovebox under N_2 or Ar (O_2 and H_2O <1 ppm) or by using standard Schlenk techniques under Ar (BOC pureshield) passed through a column containing BASF R3-11(G) catalyst and activated molecular sieves (4 Å). All glassware was dried at 160 °C overnight prior to use. Filter cannulas were prepared using Whatman 25 mm glass microfiber filters and were pre-dried at 160 °C overnight. THF and toluene were dried over molten K and distilled under a N_2 atmosphere and were kept in Young ampules over activated molecular sieves (4 Å) or a potassium mirror, respectively, under Ar. Hydrocarbons, ^{t}BuOMe and Et_2O were dried over NaK, distilled under a N_2 atmosphere, and kept in Young ampules over a potassium mirror or activated 4 Å molecular sieves (Et_2O) under Ar. $SiMe_4$ (99%) was purchased from Aldrich, degassed by three freeze−thaw cycles and dried by stirring over NaK alloy for three days before being vacuum distilled and kept over molecular sieves (4 Å) in an Ar glovebox at -35 °C. Deuterated toluene, benzene, THF and dioxane were degassed by three freeze−thaw cycles, dried by refluxing over K for 3 days, vacuum distilled, and kept in Young ampoules in the glovebox under N_2. [U{η^8-C_8H_6(1,4-Si^{i}Pr$_3$)$_2$}(η^5-Cp*)(THF)} (**1**),[i] [U{η^8-C_8H_6(1,4-$SiMe_3$)$_2$}(η^5-Cp*)] (**2**),[ii] {U[η^8-C_8H_6(1,4-Si^{i}Pr$_3$)$_2$](η^5-Cp*)Cl} (**8**)[iii] were prepared according to literature. NaN_3 (>99%) was purchased from Aldrich, dried overnight at 150 °C under dynamic high vacuum (10^{-7} mbar) and stored in a glovebox. Isocyanides and ^{t}BuNC were purchased from Aldrich, freeze-thaw-degassed (x3) and kept over 4 Å molecular sieves in Young's ampoules. ^{1}H, ^{29}Si{^{1}H}, and ^{23}Na{^{1}H} NMR data were recorded on a Varian VNMR S400 spectrometer operating at 400 MHz (^{1}H). The spectra were referenced internally to the residual protic solvent (^{1}H), or externally relative to $SiMe_4$ (^{29}Si{^{1}H}) and NaCl in D_2O (^{23}Na{^{1}H}). All spectra were recorded at 30 °C unless otherwise stated. EI-MS mass spectra were recorded on a VG-Autospec Fisons instrument at the University of Sussex unless otherwise stated. In-situ IR spectroscopy was performed using a Mettler-Toledo Autochem ReactIR 15 instrument. Elemental analyses were performed at the Microanalysis Service of the School of Chemistry at University of Bristol.

Synthesis of [U{η^8-C_8H_6-(1,4-Si^{i}Pr$_3$)$_2$}(η^5-Cp)O] (3):* In an Ar glove-box, a Young's ampoule was charged with 620 mg (0.72 mmol) [U{η^8-C_8H_6(1,4-Si^{i}Pr$_3$)$_2$}(η^5-Cp*)(THF)} (**1**). This was dissolved in *ca* 15 mL benzene with vigorous stirring to give a dark black-brown solution which was treated at RT with 94 µL of ^{t}BuNCO (d = 0.868 mg/ mL, 0.82 mmol, 1.1 mol eq), resulting in a colour change to dark brown-red. The reaction mixture was allowed to stir for 2 hours and volatiles were removed in vacuum slowly with the help of a 45-55 ºC water bath and the brown residue dried. This was dissolved in *ca* 10 mL of n-C_5 and the solution placed in a lukewarm water bath (35-45 ºC) before carefully evacuating volatiles over *ca.* 10 min while maintaining the temperature of the water bath. This was repeated three times. The resulting solid was dissolved in TMS_2O with gentle heating and filtered hot. The volume of this solution was slowly reduced under vacuum to *ca.* 1-2 mL upon which time crystals started forming. It was placed in a -45 ºC freezer overnight before the crystals were isolated by filtration and washed with cold (-80 ºC) nC_5 to give a first crop of the title compounds. The combined washings and mother-liquor produced a second crop upon cooling at -35 ºC in a glovebox freezer, while a third crop was obtained by removing the volatiles of the supernatant of the second crop, re-dissolving in $SiMe_4$/nC_5 (*ca* 2:1 ~2-3 mL), slowly evaporating in an Ar box (*ca* 1mL) and refrigerating at -35 ºC. Crystals suitable for X-ray diffraction were grown from a saturated solution of (**3**) in $SiMe_4$/nC_5. Combined yield: 320 mg (55%). ^{1}H-NMR δ(C_6D_6): -6.18 (br s, 18H, CH(C*H$_3$*)$_2$), -5.04 (br

s, 6H, C*H*(CH_3)$_2$), 0.71 (br s, 18H, CH(C*H*$_3$)$_2$), 2.54 (br s, 15H, C_5*Me*$_5$); ^{29}Si{^{1}H}-NMR δ(C_6D_6): -72.7 (s, *Si*^{i}Pr$_3$); EI-MS: 806 (M), 671 (M-Cp*), 135 (Cp*), 72 ($SiMe_3$); Elem. Anal: Calcd. for $C_{36}H_{43}OSi_2U$.0.3($SiMe_3$)$_2$O: C 53.06, H 8.09; Found: C 52.97, H 7.71.

<u>*Reaction of (3) with ^{t}BuNC:*</u> 28 mg (0.035 mmol) of (**3**) were dissolved in a Young's NMR tube and the solution was treated with 4 μL of ^{t}BuNC at RT administered via a microsyringe and the NMR spectra collected. ^{1}H-NMR (δ C_6D_6): -5.48 (2 overlapping broad s., 18H, CH(C*H*$_3$)$_2$), -4.40 ppm (br. s., 6H C*H*(CH_3)$_2$), 0.76 and 0.87 (2 overlapping singlets, 27H, CH(C*H*$_3$)$_2$ and (C*H*$_3$)$_3$C-NC), 2.59 (br. s., 15H C_5*Me*$_5$); ^{29}Si{^{1}H}-NMR δ(C_6D_6): -72 (s, *Si*^{i}Pr$_3$).

<u>*Reaction of [U{η^8-C$_8$H$_6$(1,4-SiMe$_3$)$_2$}(η^5-Cp*)] (2) with ^{t}BuNCO:*</u> A Young's NMR tube was charged with 30 mg (0.043 mol) of (**2**) that were dissolved in C_6D_6. To this brown-olive green solution was added *via* microsyringe 5.5 μL (1.1 mol eq) of ^{t}BuNCO resulting in an immediate colour change to bright red.

<u>*Independent Synthesis of {U[η^8-C$_8$H$_6$(1,4-Si^{i}Pr$_3$)$_2$](η^5-Cp*)}$_2$(μ-O) (5) from (1) and N$_2$O*</u>: 210 mg (0.245 mmol) of (**1**) were dissolved in d$_8$-toluene (*ca.* 1 mL) in a 50 mL Young's ampule with a capillary sidearm, connected to a Töpler line and degassed at -78 ºC for 5 minutes. This solution was treated with exactly 0.5 mol eq of N_2O at this temperature with stirring and the reaction was left to equilibrate at RT over 2 hours to produce an intense red solution. ^{1}H-NMR spectroscopy showed conversion to (**6**) in *ca.* 90%. Volatiles were removed in vacuum and the red residue was dissolved in ^{t}BuOMe (*ca.* 5 mL) and upon cooling at 5 ºC produced crystals of the title compound as the ^{t}BuOMe solvate that were isolated by filtration and dried in vacuum. Yield: 140 mg (79.1%). Spectroscopic data were identical to the ones previously reported. NOTE: If an excess of N_2O is used, the reaction yields brown green solids that are insoluble in organic solvents.

<u>*NMR scale reaction of (3) with (1):*</u> A Young's NMR tube was charged with 15 mg (0.0186 mmol) of (**3**) and 16 mg (1 mol eq) of (**1**) and the two solids were dissolved in C_6D_6 and the NMR spectra recorded.

<u>*Reaction of (1) with SiMe$_3$NCO (7):*</u> 25.7 mg (0.030 mmol) of (**1**) were dissolved in C_6D_6 in a Young's NMR tube and were treated at RT with 4 μL (1 mol eq, d = 0.859 mg/μL) to yield almost instantly a deep red solution. The spectra were recorded and are identical to those reported below for the independent synthesis of (**7**).

<u>*Synthesis of NaOSiMe$_3$*</u>: A suspension of NaH (723 mg, 30.1 mmol) in toluene (20 mL) placed in a high pressure ampoule was treated with a solution of 2.26 g (25.1 mmol) of degassed and dried overnight over 4Å molecular sieves Me_3SiOH (purchased from Aldrich) in toluene (20 mL) dropwise at 0 °C over *ca.* 30 min. After the addition was complete the reaction mixture was let to warm at RT before being placed under partial vacuum and heated at 85 °C in a thermostated oil bath overnight. It was then filtered *via* canulla and the volatiles removed to afford a gummy solid. This was dissolved in the minimum amount of n-pentane (5-10 mL) and refrigerated at -45 °C over 48 hrs before the crystals were isolated by filtration at -80 °C, washed with cold (-80 °C) n-pentane (2 x 5 mL) and dried in vacuum. Yield: 1.2 g (50%). ^{1}H-NMR (δ C_6D_6): 0.17 (s, 9H, Si*Me*$_3$); ^{29}Si{^{1}H}-NMR (δ C_7D_8): -5.11 (s, *Si*Me$_3$).

<u>*Independent synthesis of [U{η^8-C$_8$H$_6$(1,4-Si^{i}Pr$_3$)$_2$}(η^5-Cp*)(OSiMe$_3$)] (7)*</u>: [U{η^8-C$_8$H$_6$(1,4-Si^{i}Pr$_3$)$_2$}(η^5-Cp*)Cl} (**8**) and NaOTMS were placed in a Young's ampoule in an Ar glovebox and THF (*ca* 5 mL)

was added at RT and the mixture let to stir for 30 minutes. Volatiles were removed in vacuum and the bright red residue was dried in vacuum. It was taken in $SiMe_4$ (*ca* 10 mL) before being filtered through a small plug of Celite in a filter pipette. The solution was let to evaporate slowly to almost dryness before being placed at -35 ºC to produce the title compound as red crystals. ^{1}H-NMR (δ C_6D_6): -75.18 (s, 2H, C*H*), -43.45 (s, 2H, C*H*), -16.30 (s, 6H, C*H*(CH_3)$_2$), -13.32 (d, $^3J_{HH}$ = 5.72 Hz, 18H, CH(CH_3)$_2$), -9.38 (d, $^3J_{HH}$ = 7.63 Hz, 18H, CH(CH_3)$_2$), -4.49 (s, 15H, C_5(CH_3)$_5$), 30.32 (s, 9H, Si(CH_3)$_3$), 95.74 (s, 2H, C*H*); ^{29}Si{^{1}H}-NMR (δ C_6D_6): -128.81 (the other Si environment-presumably the $OSiMe_3$ one- could not be located); EI-MS: 879 (M), 744 (M-Cp*), 461 (UCp*$OSiMe_3$), 157 ((Si^{iPr3})$_3$), 115 (H(Si^{iPr3})$_2$); Elem. Anal.: Calcd for $C_{39}H_{72}OSi_3U$: C 53.27; H 8.25. Found: C 53.58; H 7.93.

Discussion of the molecular structure of (7):

Crystals suitable for single crystal XRD were obtained from the absolute minimum of $SiMe_4$ (*ca.* 0.5 mL). An ORTEP diagram of its molecular structure is presented in **Figure SI1**.

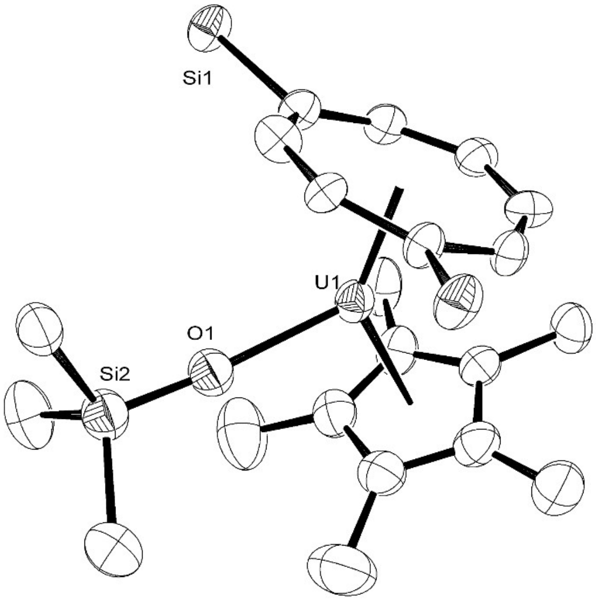

Figure SI1: ORTEP diagram of the molecular structure of (**7**) displaying 50% probability ellipsoids Hydrogen atoms and ^{i}Pr groups have been omitted for clarity. Selected bond lengths (Å) and angles (º): O1-Si2 1.55(2), O1-U1 2.215(18), Ct(COT)-U1 1.953(8), Ct(Cp*)-U1 2.485(2); Si2-O1-U1 173.8(12), Ct(COT)-U1-Ct(Cp*) 135.21(6).

The only salient feature of this molecular structure is the rather long U-O bond distance compared to [U{OSi(Mes)$_3$}$_3$]$_2$(μ-η^1:η^1-N_2),[iv] [U{$OSiMes_3$}$_4$],[iv] U{Cp^{3T}}$_2$($OSiMe_3$)CN (Cp^{3T} = (C_5H_2)^{t}Bu$_3$),[v] UCp_3OSiPh_3,[vi] [U{OSi(Mes)$_3$}{N($SiMe_3$)$_2$}$_3$],[iv] and [U{OSi(O^{t}Bu)$_3$}$_2${μ-OSi(O^{t}Bu)$_3$}]$_2$.[vii] A possible reason for this is to reduce the steric congestion due to the bulky Si^{i}Pr$_3$ substituted COT ligand. All other metric features are as expected and warrant no further discussion.

*Synthesis of [U{η^8-C_8H_6(1,4-Si^{i}Pr$_3$)$_2$}(η^5-Cp*)NNa(OEt_2)$_2$](**9**):* 320 mg (0.38 mmol) of (**1**) and 26 mg (1.02 mol eq) of NaN_3 were charged in a Young's ampule in an Ar box and toluene and THF was

added to the solids (*ca.* 2:1 v/v 10 mL total) at RT and the reaction mixture was stirred overnight. Volatiles were removed in vacuum to leave a brown-red residue that was extracted in warm (almost boiling) Et_2O filtered hot (3x5 mL) followed by extraction and filtration of the remaining residue with hot toluene (not boiling) (*ca* 5 mL). Upon standing at RT the filtrate starts depositing crystals. The volume was slowly reduced to *ca.* 5 mL and refrigerated at -45 ºC overnight to complete crystallisation. The crystals were isolated by filtration cold -45 ºC and washed with cold (-45 ºC) nC_5 and dried in vacuum to yield the title compound. A second crop (*ca* 10-20 mg) was isolated by refrigeration (-45 ºC) of the combined wash and mother-liquor but was not as pure as the first one. Crystals suitable for X-ray diffraction were grown by slow evaporation of a saturated solution of (**9**) in Et_2O. Analytically pure samples were obtained from recrystallization from hot dioxane. Yield (1st crop): 90 mg (24.3%). ^{1}H-NMR δ(C_4D_8O): -7.63 (br. s., 18H, CH(C*H₃*)₂), -2.10 and -1.10 (overlapping br. s., 24H, C*H*(C*H₃*)₂), 1.08 (t, $^{3}J_{HH}$ = 6.68 Hz, 12H, (C*H₃*CH₂)₂O), 3.35 (q., $^{3}J_{HH}$ = 6.68 Hz, 8H, (CH₃C*H₂*)₂O); ^{29}Si{^{1}H}-NMR (δ C_4D_8O): -70.9; ^{23}Na{^{1}H}-NMR (δ $C_4D_8O_2$): 200 ($\Delta\nu_{1/2}$ = 8300 Hz). No molecular ion could be observed. Elem. Anal: Calcd. for $C_{44}H_{83}NaNO_2Si_2U$: C 54.18, H 8.58, N 1.44; Found: C 53.78, H 8.49, N 1.55.

Synthesis of [{U[η⁸-C₈H₆(1,4-SiMe₃)₂](η⁵-Cp)}₂(μ-N)]⁻[Na(THF)ₓ]⁺ (**10/10'**):* This was prepared as in the case of (**9**) from 400 mg (0.576 mmol) of (**2**) and 20 mg (0.53 mol eq) of NaN_3 and after removal of the toluene/THF reaction solvent mixture, the brown residue was extracted in Et2O in an Ar glovebox, filtered (through a filter pipette) into a 20 mL scintillation vial (3x5 mL) and the brown residue left on the filter paper was dissolved in the minimum amount of THF (*ca.* 2 mL) and combined with the Et_2O extract leaving behind an off-grey residue which was discarded. The combined THF/Et_2O extract was reduced slowly in volume at RT using an Ar stream (*ca* 2-3 mL) until crystalline material started depositing. It was refrigerated overnight (-35 ºC) before the crystals were carefully separated from the mother-liquor using a drown-out pipette, washed with nC_5 and dried in vacuum to yield (**10'**) as a brown crystalline powder. A second crop can be isolated by slow evaporation followed by refrigeration of the combined nC_5 wash and supernatant. X-ray quality crystals of (**10**) can be isolated by refrigeration of a saturated solution (*ca* 1 mL) of (**10'**) in Et_2O with a 1-2 drops of THF. Combined yield: 335 mg (81 %). ^{1}H-NMR δ($C_4D_8O_2$): -3.46 (s, 18H, Si(C*H₃*)₃), 0.55 (s, 18H, (C*H₃*)₃), 1.76 (br s, 8H, (C*H₂*CH₂)₂O), 2.58 (br. s, 30H, C₅(C*H₃*)₅), 3.62 (br m, 8H, (CH₂C*H₂*)₂O); ^{29}Si{^{1}H}-NMR (δ $C_4D_8O_2$): -98.88 and -88.22 (s, *Si*Me₃); ^{23}Na{^{1}H}-NMR (δ $C_4D_8O_2$): -7.94 ($\Delta\nu_{1/2}$ = 78 Hz). No molecular ion could be observed. Elem. Anal: Calcd. for $C_{56}H_{94}NaNO_2Si_4U_2$ (x = 2; **10'**): C 47.21, H 6.65, N 0.98; Found C 46.97, H 6.59, N 1.01.

Synthesis of [U{η⁸-C₈H₆(1,4-SiⁱPr₃)₂}(η⁵-Cp) O]⁻[K(18-crown-6)]⁺(**11**)***:** An ampule was charged with 192 mg (0.238 mmol) of (**3**) and 63 mg of 18-crown-6 (1 mol eq) and nC_5 (*ca.* 10 mL) followed by Et_2O (*ca.* 2 mL) was added to the solids with vigorous stirring. To this solution were added *via* pipette 2.2 g (1.2 mol eq) of K/Hg (0.5% w/w) and after a few minutes a pink-red solid formed. The reaction mixture was stirred for another 2 hrs at RT before it was filtered and the solids were washed once with nC_5. To this residue was added toluene (5-10 mL) and was gently heated my means of a heat-gun until (with the aid of stirring) all the pink-red residue had produced a deep red solution with insoluble Hg at the bottom. It was filtered while hot and the filtrate was slowly reduced in vacuum to produce deep red rods of the title compound that were also suitable for single crystal XRD studies. They were isolated by careful decantation of the mother-liquor and washed once with toluene (*ca.* 1 mL), then nC_5 (2 pipette-fulls) and finally Et_2O (1 pipette full) before drying in vacuum. Yield: 150 mg (52.3%). ^{1}H-NMR δ(C_4D_8O): -21.65 (s, 6H, C*H*(CH₃)₂), -17.98 (s, 18H, CH(C*H₃*)₂), -9.87 (br s, 15H, C₅Me₅), -5.27 (s,

18H, CH(CH_3)$_2$), 11.56 (s, 24H, CH_2) the signals for the COT protons could not be located; ^{29}Si{^{1}H}-NMR δ(C_4D_8O): -172.22 (s, *Si*^{i}Pr$_3$); No molecular ion could be observed; Elem. Anal: Calcd. for $C_{48}H_{87}KO_7Si_2U$: C 51.96, H 7.90; Found C 51.63, H 7.80 (crystals of (**11**) loose C_7H_8 under vacuum with concurrent loss of crystallinity).

SQUID MAGNETOMETRY:

Magnetic measurements of polycrystalline (**10'**) were carried out using a Quantum Design MPMS-5 SQUID magnetometer at 0.1 Tesla in the range 2 – 300 K, and for (**9**) and (**3**) using a Quantum Design MPMS-*XL* SQUID magnetometer at 0.1 Tesla in the range 5 – 300 K. The accurately weighed samples (20 – 40 mg) were placed into a gelatine capsule and then loaded into a nonmagnetic plastic straw before being lowered into the cryostat. Values of the magnetic susceptibility were corrected for the underlying diamagnetic increment by using tabulated Pascal's constants,[viii] and the effect of the blank sample holders (gelatin capsule/straw).

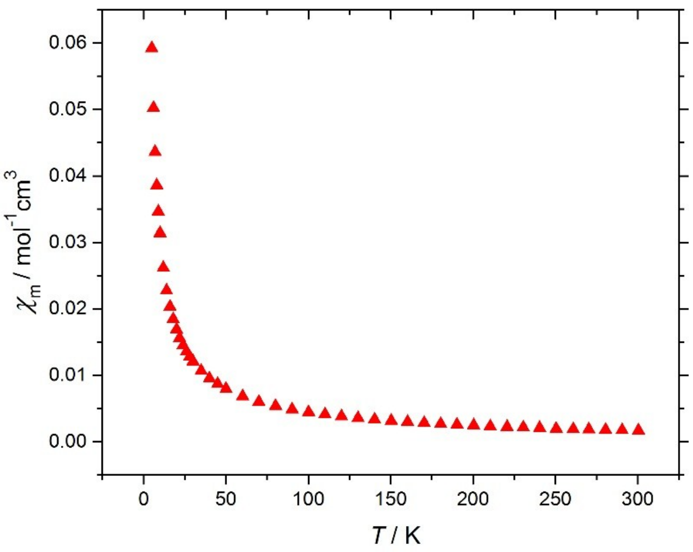

Figure SI2: Temperature dependence of the solid state susceptibility χ_m of (**3**) at 0.1 Tesla.

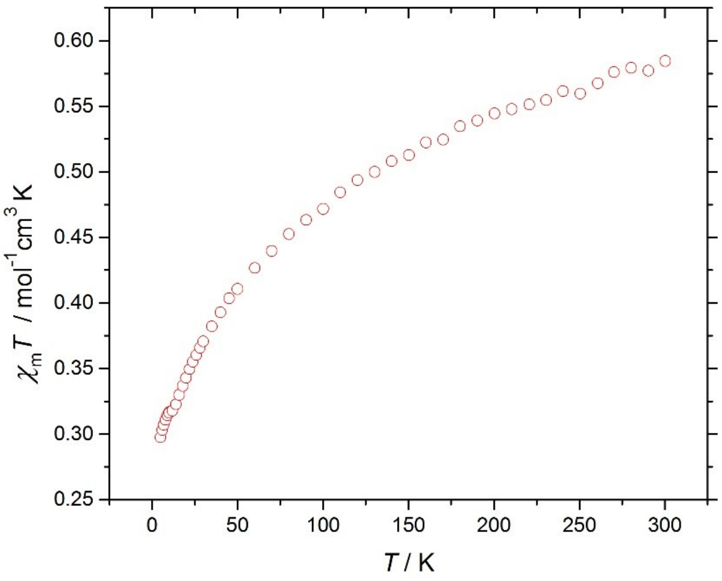

Figure SI3: Temperature dependence of the solid state $\chi_m T$ product of (**3**) at 0.1 Tesla.

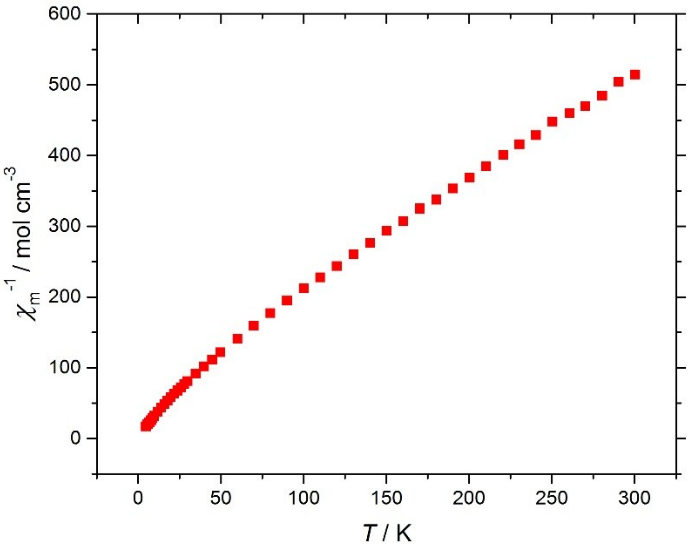

Figure SI4: Temperature dependence of the inverse susceptibility χ_m^{-1} of (**3**) at 0.1 Tesla.

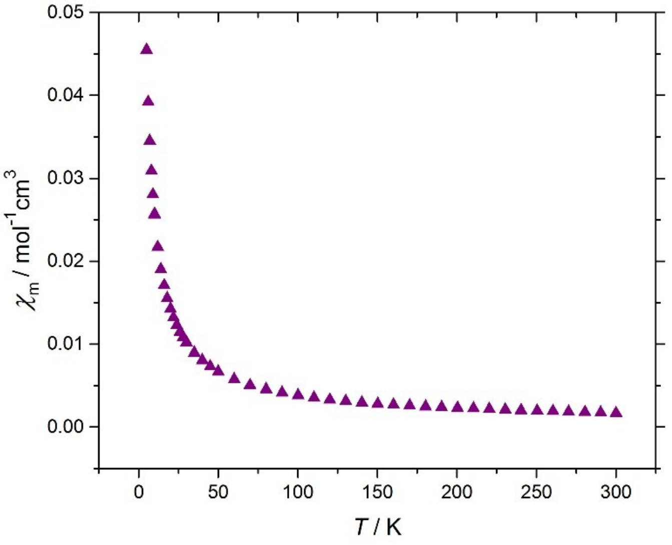

Figure SI5: Temperature dependence of the solid state susceptibility χ_m of (**9**) at 0.1 Tesla.

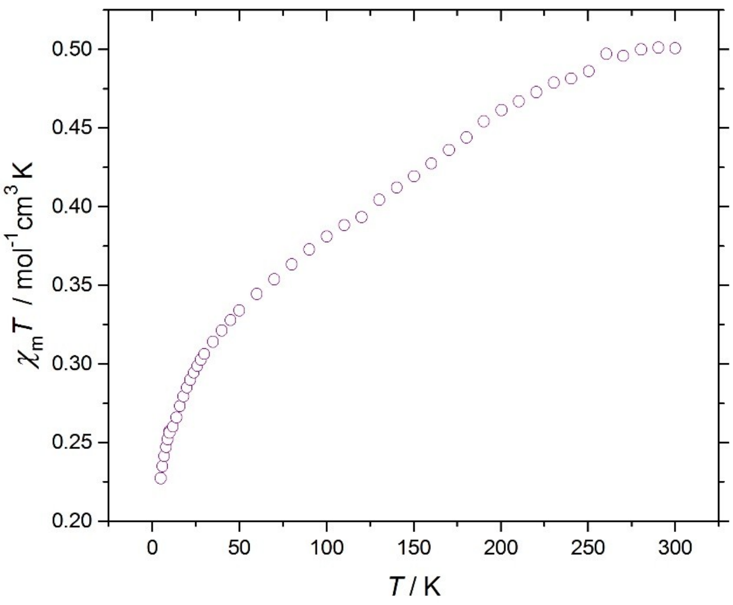

Figure SI6: Temperature dependence of the solid state $\chi_m T$ product of (**2**) at 0.1 Tesla.

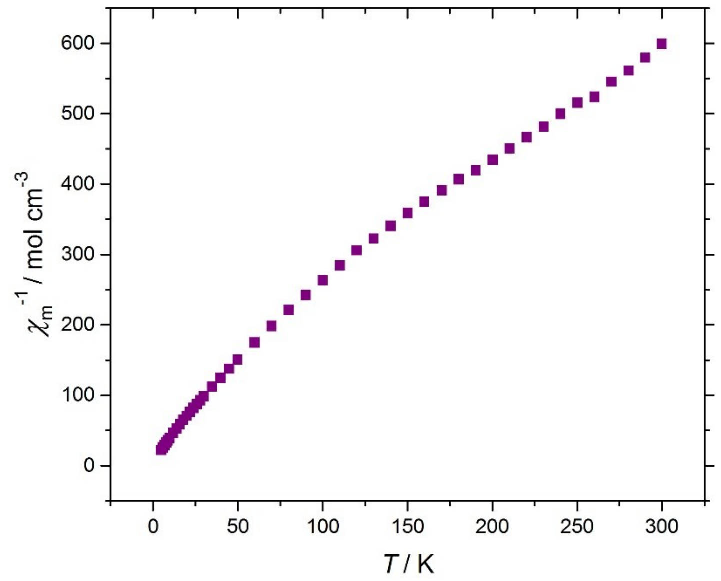

Figure SI7: Temperature dependence of the inverse susceptibility χ_m^{-1} of (**9**) at 0.1 Tesla.

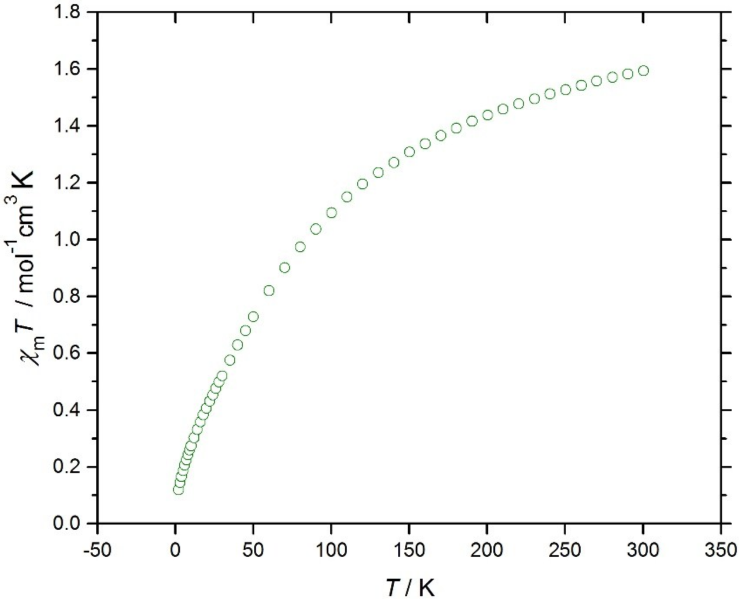

Figure SI8: Temperature dependence of the solid state $\chi_m T$ product of (**10'**) at 0.1 Tesla.

Cyclic Voltametry:

Cyclic Voltametry studies were performed in an Ar glovebox using a BASi-Epsilon potentiostat under computer control. IR drop was compensated using the feedback method. CV experiments were performed using the three electrode method with glassy carbon disk (7.0 mm^2) as the working electrode, Pt wire as the counter electrode and Ag wire as the pseudoreference electrode. Sample

solutions were prepared by dissolving the appropriate supporting electrolyte in 1mL of solvent followed by addition of the analyte to give a concentration of *ca* 5mM. The reported half potential are referenced to $Fc^{0/+}$ redox couple, which was measured by adding ferrocene (*ca* 1mg) to the sample solution. In the case of (**10'**) no processes could be observed using THF and either $[N(^nBu)_4][B(C_6F_5)_4]$ or $[N(^nBu)_4]PF_6$ as the supporting electrolyte or by using Au or Pt as the working electrode. When CH_3CN (dried over CaH_2 under an Ar, distilled and kept over activated 3 Å molecular sieves) employed as a solvent and $[N(^nBu)_4]PF_6$ as electrolyte we managed to observe current responses upon scanning voltages over the solvent window. It has to be noted though that (**10'**) reacts slowly with CH_3CN ($t_{1/2}$ of approximately 3 hours) and as a result the experiment must be performed as quickly as possible. The product of this reaction is currently under investigation. In the case of (**1**) we used a similar procedure for the reasons previously described and its voltammogram had the same features as the ones observed for complexes of the type $[U\{\eta^8\text{-}C_8H_6(1,4\text{-}SiMe_3)_2\}(\eta^5\text{-}Cp^{Me4R})THF]$ (R = Me, Et, ^{i}Pr, ^{t}Bu).

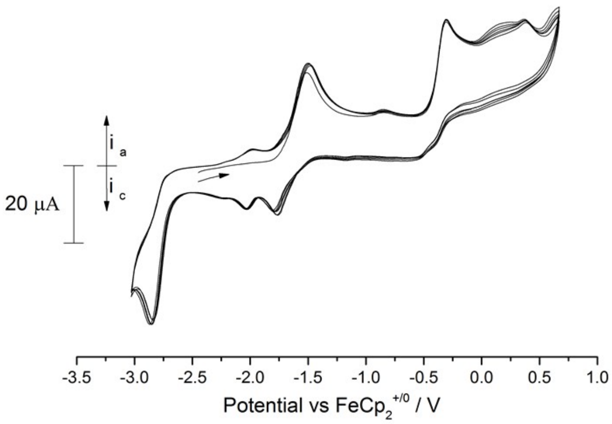

Figure SI9: Overlaid CV scans (3 cycles) of (**9**) in 0.05 M $[N(^nBu)_4][B(C_6F_5)_4]$/THF showing the additional processes observed. Scan rate 100 mV s^{-1}.

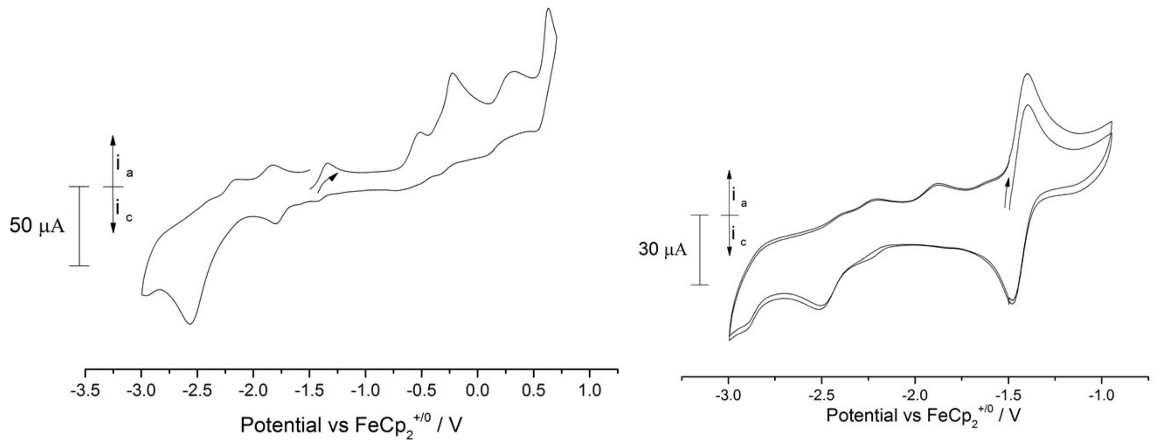

Figure SI10: From left to right (a) CV scan of (**10'**) showing the additional processes observed; (b) overlaid CV scan of (**10'**) between -3.0 and -1.0 V. Both in 0.1 M $[N(^nBu)_4]PF_6$/MeCN and using a scan rate of 300 mV.s^{-1}.

As can be seen from Figure SI9 another reversible process at *ca* -1.8 V is present that disappears when a smaller window is scanned. We tentatively assign this process to an electrochemically generated species. More in depth analysis was not possible due to the instability of (**10’**) in MeCN.

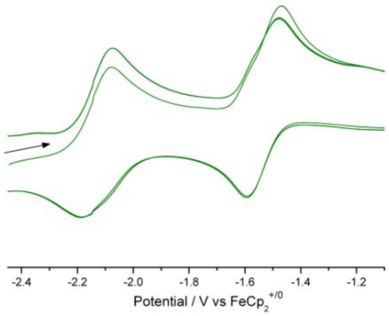

Figure SI11: Overlaid CV scans of (**1**) in 0.05M $[N(^nBu)_4][B(C_6F_5)_4]$/THF. Scan Rate 200 mV.s^{-1}.

Table SI1: Electrochemical parameters for **3**, **9** and **10**.

**Compound**	**E_{pa} / mV**	**E_{pc} / mV**	**ΔE_{pp} / mV**	**i_{pa} / µA**	**i_{pc} / µA**	**i_{pc}/i_{pa}**
**3**	-464	-573	109	47.01	51.97	1.11
**9**	237	-51	288	41.06	24.96	0.61
**10**	83	-4	87	33.81	34.64	1.02

Potentials measured vs. $Ag^{+/0}$ pseudo-reference electrode. $\Delta E_{pp} = | E_{pc} - E_{pa} |$

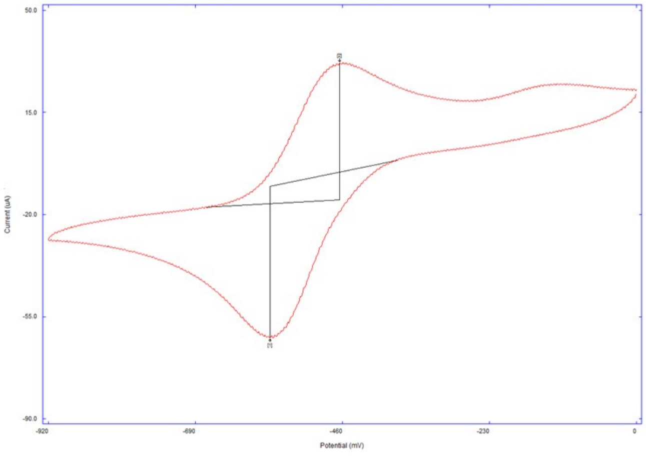

Figure SI12: Determination of peak potentials and currents for 3.

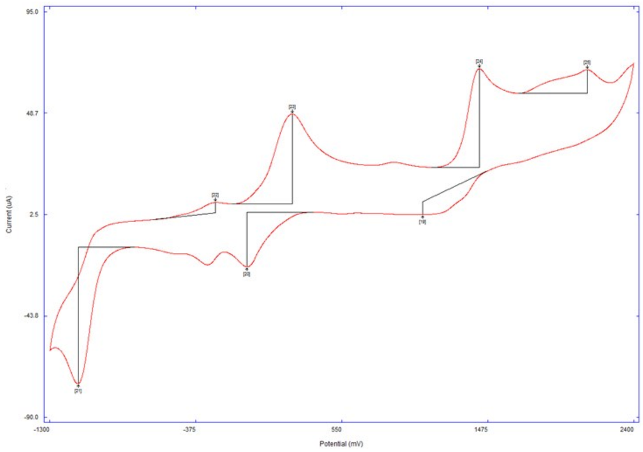

Figure SI13: Determination of peak potentials and currents for 9.

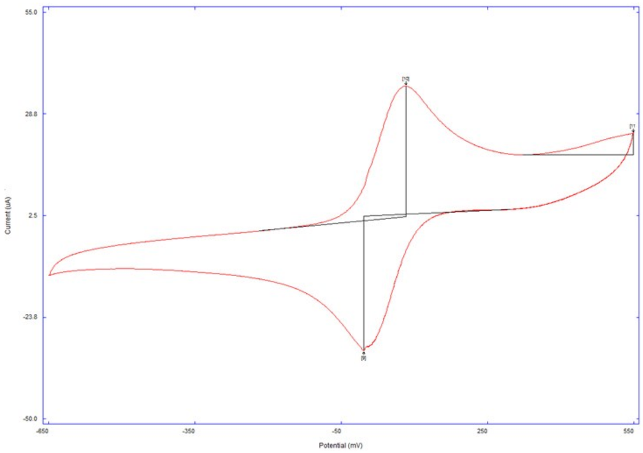

Figure SI14: Determination of peak potentials and currents for 10.

UV-Visible Spectroscopy: For (**3**) two absorptions at 392 nm (ε_{max} = 3515.7 $cm^{-1}.M^{-1}$) and 463 nm (ε_{max} = 2270.4 $cm^{-1}.M^{-1}$); for (**9**) two absorptions at 388 nm (ε_{max} = 2455.7 $cm^{-1}.M^{-1}$) and 478 nm (ε_{max} = 2193.4 $cm^{-1}.M^{-1}$); for (**10**) no discernible absorptions could be detected in the region scanned before the onset of the LMCT band. In the range of 210-300 nm all complexes exhibited strong absorptions attributed to LMCT bands, resulting in saturation of the detector even in very low concentrations (2-5 mM).

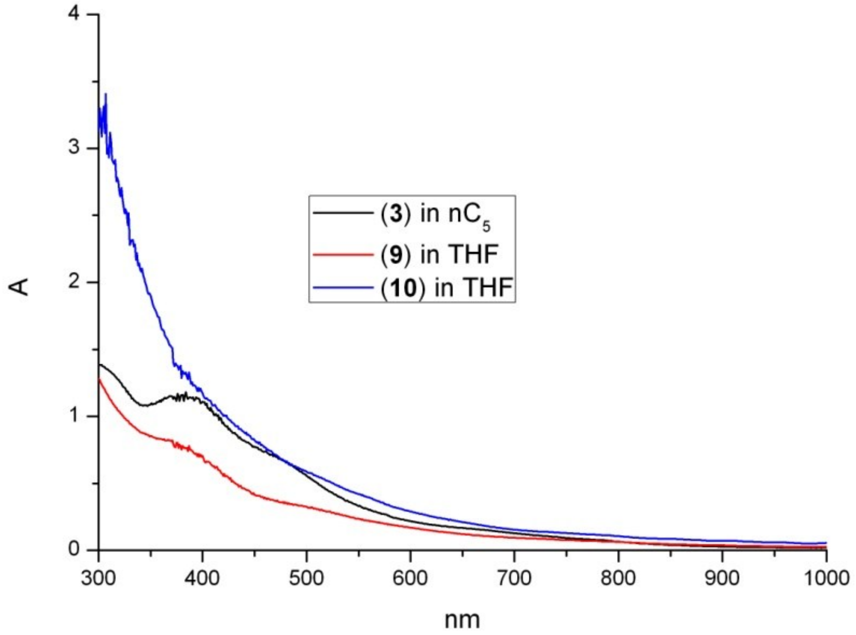

Figure SI15: UV-VIS Spectra of (**3**), (**9**) and (**10**)

^{23}Na NMR Spectra :

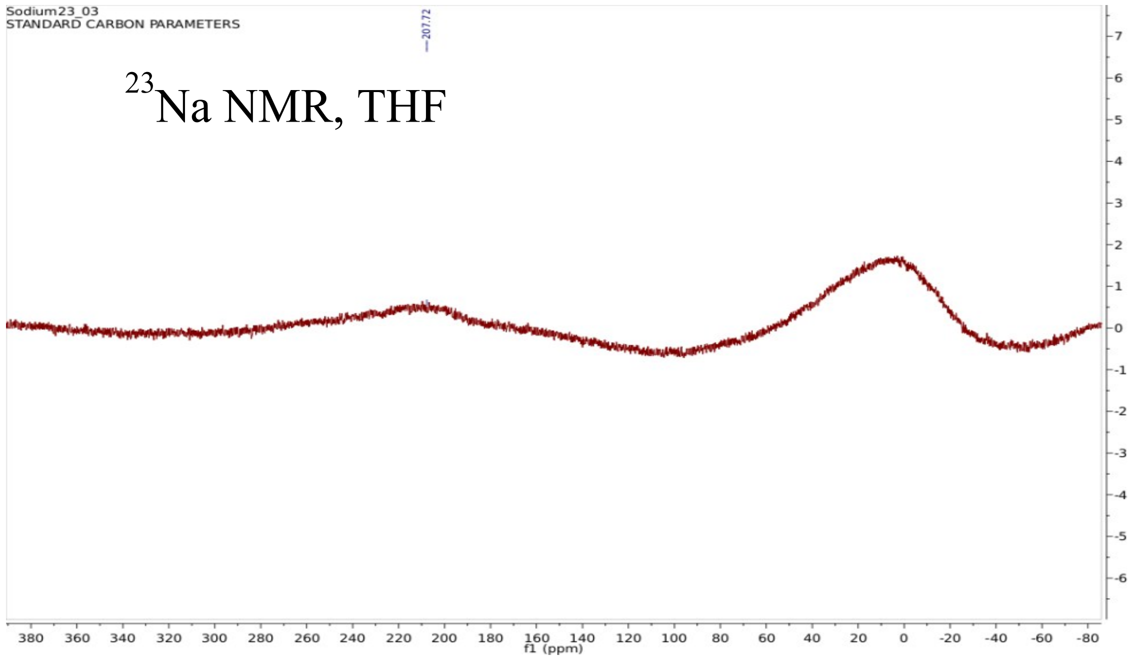

Figure SI16: ^{23}Na NMR Spectrum of (**9**)

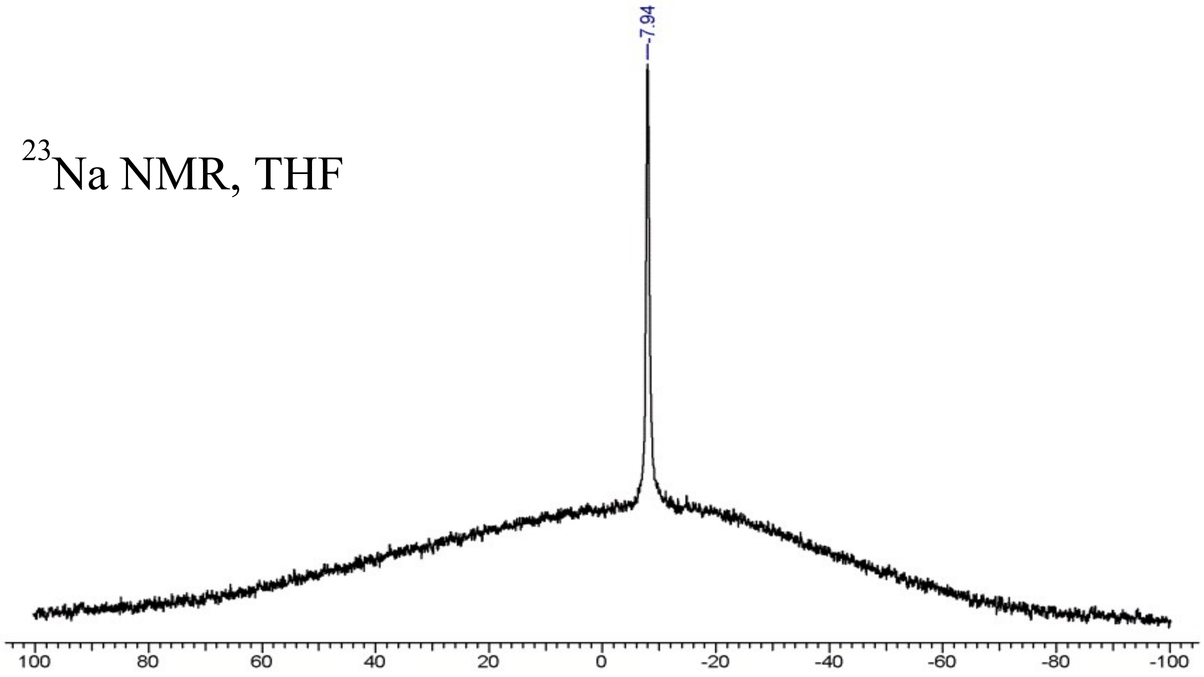

Figure SI17: ^{23}Na NMR Spectrum of (**10**)

X-ray crystallography: Datasets for all complexes herein were collected using an Agilent Gemini Ultra diffractometer with an Enhance (Mo *Kα*) (for (**3**), (**4**) and (**11**)) or an Enhance Ultra source (Cu *Kα*) (for (**7**), (**9**) and (**10**)) source, equipped with an Eos CCD area detector and an Oxford Cryosystems low-temperature device (173 K), operating in ω scanning mode with ψ and ω scans to fill the Ewald sphere. Control, integration and absorption correction were handled by the CrysAlis Pro software. The crystals were mounted on MiTiGen loops, from dried vacuum oil kept over 4Å in a MBraun glovebox under Ar. All solutions and refinements were performed using the WinGX package and all software packages within. All non-hydrogen atoms were refined using anisotropic thermal parameters, and hydrogens were added using a riding model. Crystal structure, data collection and refinement details are given Table SI1. CCDC numbers1449997-1450002 for compounds **(3)**, **(4)**, **(7)**, **(9)**, **(10)**, and **(11)**

Compound	(**3**)	(**4**)	(**7**)	(**9**)	(**10**)	(**11**)
Colour, Habit	Brown-red, Plate	Brown, Irregular	Red, Plate	Brown, Plate	Brown, Irregular	Red, Rod
Size/mm	0.15x0.15x0.08	0.2x0.1x0.0 5	0.2x0.1x0.05	0.25x0.15x0. 03	0.15x0.12x0. 08	0.30x0.1x0.05
Empirical Formula	$C_{36}H_{63}OSi_2U$	$C_{41}H_{72}NOSi_2U$	$C_{39}H_{72}OSi_3U$	$C_{44}H_{83}NaNO_2Si_2U$	$C_{72}H_{126}NNaO_6Si_4U_2$	$C_{43}H_{87}KO_6Si_2U.C_7H_8$
M	806.7	889.2	879.26	975.31	1485.33	600.81
Crystal System	Monoclinic	Monoclinic	Orthorhombic	Monoclinic	Triclinic	Monoclinic
Space Group	*C2/c*	$P2_1/c$	*Pnma*	$P2_1/m$	*P*-1	$P2_1/m$
a/Å	38.0927(19)	22.8816(13)	16.6069(13)	10.7251(2)	12.3696(4)	11.1168(4)
b/Å	11.6377(6)	12.0603(5)	22.4849(19)	18.7626(3)	13.4214(5)	19.7384(6)
c/Å	16.7259(7)	16.2087(8)	11.2503(11)	11.9516(2)	21.7314(6)	13.0100(5)
α/°	90	90	90	90	98.997(3)	90
β/°	100.791(5)	108.372(6)	90	95.2380(10)	99.477(2)	90.901(3)
γ /°	90	90	90	90	109.711(3)	90
V/ $Å^3$	7283.7(6)	4245.0(4)	4200.9(6)	2394.99(7)	3261.6(2)	2854.40(17)
Z	8	4	4	4	2	4
μ/mm^{-1}	4.548	3.909	11.880	10.344	14.900	3.005
T (K)	173(2)	173(2)	173(2)	173(2)	173(2)	173(2)
θmin/max	3.236/26.372	3.378/26.19 9	9.733/67.079	3.714/71.569	3.593/70.167	3.6430/27.9870
Completeness	99.8 to 26.372	83.9 to 26.199	98.9 to 67.079	97.4 to 71.569	96.1 to 70.167	99.8 to 27.98
Reflections Total/Independent	7446/6027	9665/7542	3802/2307	4694/4318	11925/9505	6004/ 5357
R_{int}	0.0495	0.0594	0.1458	0.0728	0.0448	0.0604
Final *R1* and *wR2*	0.0344 and 0.0705	0.0594 and 0.1376	0.0983 and 0.2983	0.0305 and 0.0762	0.0388 and 0.0908	0.0371 and 0.0744
Larget peak hole/ e.$Å^{-3}$	1.065 and -0.763	1.544 and - 1.006	1.595 and - 2.345	1.540 and - 1.381	1.386 and - 0.856	1.454 and -1.438
ρ_{calc}/g.cm^{-3}	1.470	1.391	1.390	1.352	1.460	1.462

Table SI 2: X-Ray Data

[i] O. T. Summerscales, F. G. N. Cloke, P. B. Hitchcock, J. C. Green and N. Hazari, *Science,* 2006, **311,** 829.
[ii] N. Tsoureas, O. T. Summerscales, F. G. N. Cloke and S. M. Roe, *Organometallics,* 2013, **32,** 1353.
[iii] J. A. Higgins, F. G. N. Cloke and S. M. Roe, *Organometallics,* 2013, **32,** 5244-5252
[iv] S. M. Mansell, J. H. Farnaby, A. I. Germeroth, P. L. Arnold, *Organometallics,* 2013, **32,** 4214-4222.
[v] G. Zi, L. Jia, E. L. Werkema, M. D. Walter, J. P. Gottfriesden, R. A. Andersen, *Organometallics,* 2005, **24,** 4251-4264
[vi] M. Porchia, N. Brianese, U. Casellato, F. Ossola, G. Rossetto and P. Zanella, *J. Chem. Soc., Dalton Trans.,* 1989, 677-681
[vii] V. Mougel, C. Camp, J. Pécaut, C. Copéret, L. Maron, C. E. Kefalidis, M. Mazzanti *Angew. Chem. Intl. Ed.,* 2012, **51,** 12280-12284.
[viii] G. A. Bain and J. F. Berry, *J. Chem. Educ.*, 2008, **85**, 532–536.
